# mTOR–neuropeptide Y signaling sensitizes nociceptors to drive neuropathic pain

**DOI:** 10.1172/jci.insight.159247

**Published:** 2022-11-22

**Authors:** Lunhao Chen, Yaling Hu, Siyuan Wang, Kelei Cao, Weihao Mai, Weilin Sha, Huan Ma, Ling-Hui Zeng, Zhen-Zhong Xu, Yong-Jing Gao, Shumin Duan, Yue Wang, Zhihua Gao

**Affiliations:** 1 Spine Lab, Department of Orthopedic Surgery, The First Affiliated Hospital, Zhejiang University School of Medicine, Hangzhou, China.; 2 Department of Neurobiology and Department of Neurology of Second Affiliated Hospital, Zhejiang University School of Medicine, Hangzhou, China.; 3 Liangzhu Laboratory, Zhejiang University Medical Center, MOE Frontier Science Center for Brain Science and Brain-machine Integration, State Key Laboratory of Brain-machine Intelligence, Zhejiang University, Hangzhou, China.; 4 NHC and CAMS Key Laboratory of Medical Neurobiology, Zhejiang University, Hangzhou, China.; 5 Institute of Pain Medicine and Special Environmental Medicine, Nantong University, Nantong, China.; 6 Key Laboratory of Novel Targets and Drug Study for Neural Repair of Zhejiang Province, School of Medicine, Zhejiang University City College, Hangzhou, China.

**Keywords:** Neuroscience, NPY, Pain

## Abstract

Neuropathic pain is a refractory condition that involves de novo protein synthesis in the nociceptive pathway. The mTOR is a master regulator of protein translation; however, mechanisms underlying its role in neuropathic pain remain elusive. Using the spared nerve injury–induced neuropathic pain model, we found that mTOR was preferentially activated in large-diameter dorsal root ganglion (DRG) neurons and spinal microglia. However, selective ablation of mTOR in DRG neurons, rather than microglia, alleviated acute neuropathic pain in mice. We show that injury-induced mTOR activation promoted the transcriptional induction of neuropeptide Y (*Npy*), likely via signal transducer and activator of transcription 3 phosphorylation. NPY further acted primarily on Y2 receptors (Y2R) to enhance neuronal excitability. Peripheral replenishment of NPY reversed pain alleviation upon mTOR removal, whereas Y2R antagonists prevented pain restoration. Our findings reveal an unexpected link between mTOR and NPY/Y2R in promoting nociceptor sensitization and neuropathic pain.

## Graphical abstract

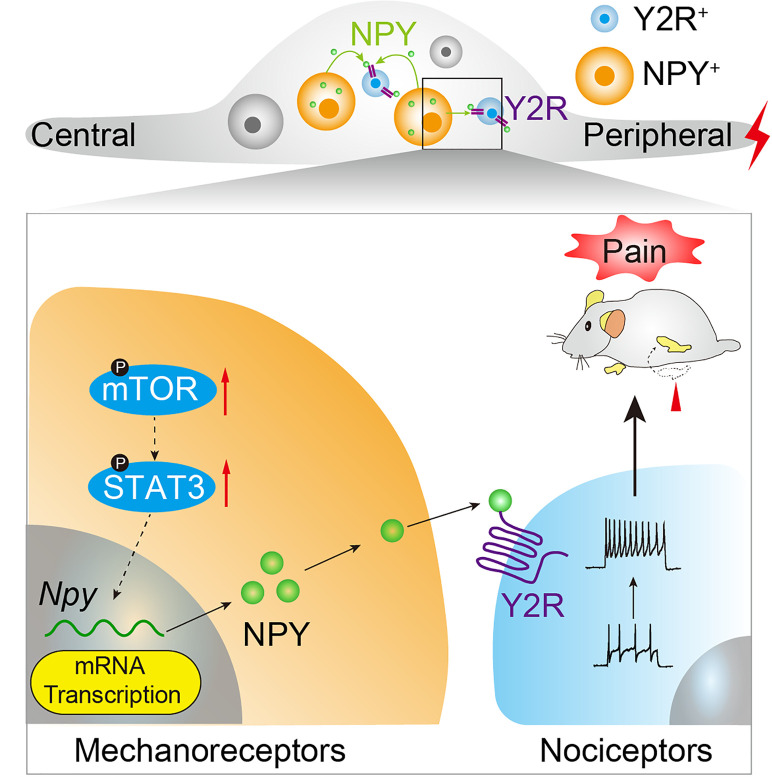

## Introduction

Chronic pain, the leading cause of long-term human disability, poses a heavy health burden to society. Nerve injury–induced neuropathic pain accounts for approximately one-fifth of the chronic pain population (1). It is characterized by persistent hyperalgesia, allodynia, and spontaneous pain. Long-lasting sensitization of the nociceptive pathway, leading to a reduced pain threshold, has been considered a major mechanism mediating the persistent hypersensitivity in neuropathic pain (2).

Accumulating evidence has shown that nerve injury–induced de novo gene expression contributes to maladaptive responses in both peripheral and central nociceptive circuits, thereby promoting nociceptive sensitization and pain hypersensitivity (2, 3). Elevation of G protein–coupled receptors (GPCRs), such as GPR151, coupled with ion channels in the injured dorsal root ganglia (DRG), has been shown to facilitate the generation of ectopic action potentials (AP) in nociceptive neurons to promote pain (4, 5).

Other than ion channels and GPCRs, prominent induction of neuropeptides, including neuropeptide Y (NPY), galanin (Gal), neurotensin (NTS), and cholecystokinin (CCK), have also been observed in DRG neurons after nerve injury (6–8). The 36–amino acid peptide, NPY, is one of the most robustly upregulated neuropeptides in DRG neurons after nerve injury (9). However, mechanisms underlying its induction remain unclear. Conditional knockdown of spinal cord NPY has been shown to increase tactile and thermal hypersensitivity primarily through Y1 receptor (Y1R) in nerve injury–induced neuropathic pain models (10, 11), whereas s.c. injection of NPY or Y2R agonist appears to exacerbate pain after nerve injury, suggesting a biphasic role of NPY in neuropathic pain at different sites (12–14). It remains to be elucidated how NPY is induced after injury and whether NPY plays opposing roles through different receptors in the nociceptive pathway.

The mTOR, a master regulator of protein translation, plays a pivotal role in regulating cell growth and metabolism. Deregulation of the mTOR signaling has been linked to various human diseases, including cancer, obesity, and neurodegeneration (15–17). Activation of mTOR has been observed in the DRG and spinal cord in neuropathic pain models and in morphine-induced chronic pain (18–21). Furthermore, pharmacologic blockade of mTOR activity has been demonstrated to reduce pain (22–27). However, several studies also noted that inhibiting mTOR complex 1 (mTORC1) resulted in unexpected mechanical allodynia, likely associated with the negative feedback activation of extracellular signal–regulated kinase 1/2 (ERK 1/2) in primary sensory neurons (3, 28). The role of mTOR in pain remains to be clarified.

Combining genetic manipulation, transcriptomic profiling, and electrophysiological recording, we uncovered a previously unrecognized link between the nerve injury–triggered mTOR activation and NPY induction in DRG neurons. We further demonstrate that mTOR-mediated NPY production enhances nociceptor excitability and promotes pain hypersensitivity through Y2R in DRGs. Although mTOR-related signaling has been extensively studied, we present the first evidence to our knowledge for mTOR-regulated NPY signaling in driving neuropathic pain development.

## Results

### Nerve injury induces mTOR activation in subsets of DRG neurons and spinal cord microglia.

**Figure 1:**
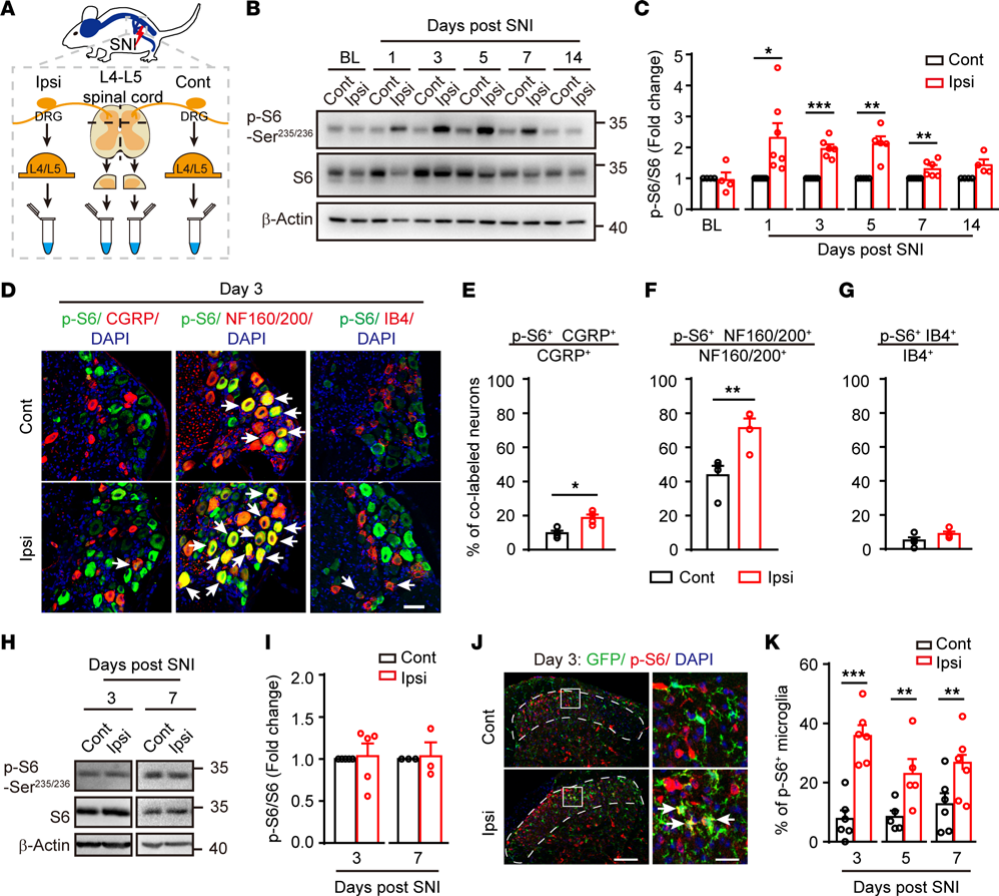
Activation of the mTOR in subsets of DRG neurons and SDH microglia after spared nerve injury (SNI). (**A**) A schematic diagram depicting the isolation of DRGs and SDH. (**B**) Representative blots indicating the upregulated p-S6 levels in the ipsilateral DRG after SNI at indicated time points. (**C**) Quantification of p-S6/S6 in the ipsilateral DRG at indicated time points after SNI (*n* = 4–7 mice per time point). (**D**) Coimmunostaining p-S6 with CGRP, NF160/200, or IB4 in DRGs after SNI (arrows indicating colabeled neurons). Scale bar: 50 μm. (**E**–**G**) Quantification of p-S6^+^ neurons in different subpopulations of DRG neurons: CGRP (**E**), NF160/200 (**F**), and IB4 (**G**) (*n* = 4 mice). (**H**) Representative blots of p-S6 and S6 levels in SDH (L4 and L5) at days 3 and day 7 after SNI. (**I**) Quantification of p-S6/S6 in the ipsilateral and contralateral SDH (*n* = 5 and 3 for day 3 and day 7 after SNI, respectively). (**J**) Representative images of p-S6^+^ microglia (arrows) in the superficial contralateral and ipsilateral SDH (dotted lines) at indicated time points after SNI. Boxes show regions of higher magnification in the SDH. Scale bars: 100 and 20 μm for low- and high-magnification images, respectively. (**K**) Quantification of p-S6^+^ microglia in superficial SDH (*n* = 5–6 mice per time point). Data are shown as mean ± SEM. *P < 0.05, **P < 0.01, and ***P < 0.001, 2-tailed paired Student’s *t* tests. BL, baseline; Ipsi, ipsilateral; Cont, contralateral; DRG: dorsal root ganglion; SDH, spinal dorsal horn.

To examine the status of mTOR activation after nerve injury, we carried out Western blot analysis of L4 and L5 DRGs and spinal dorsal horn (SDH) tissues from mice at different time points after the spared nerve injury (SNI) surgery (Figure 1A). The activity of mTOR was assessed by the levels of phosphorylated S6 protein (p-S6), a key downstream effector of mTOR. As shown in Figure 1, B and C, substantially upregulated p-S6 was found in the injured (ipsilateral) DRG at day 1 after SNI and lasted for at least 7 days (*P* < 0.05), consistent with elevated mTOR activity in DRGs after peripheral nerve injury (18).

To further determine the cellular identities with mTOR activation, we performed immunofluorescence analysis using the anti–p-S6 antibody along with different markers. Size frequency analysis showed that p-S6 was mainly present in medium and large neurons in DRG (Supplemental Figure 1A; supplemental material available online with this article; https://doi.org/10.1172/jci.insight.159247DS1). In the contralateral DRG, positive p-S6 labeling, reflecting basal mTOR activity, was observed in a small subset of CGRP^+^ peptidergic neurons (9.7%) but a large fraction of NF160/200^+^ neurons (43.7%), reminiscent of large myelinated type A fiber mechanoreceptors. In the ipsilateral DRG, a substantial increase of p-S6 labeling in NF160/200^+^ large mechanoreceptors (from 43.7% to 71.2%, *P* < 0.01) and CGRP^+^ peptidergic neurons (from 9.7% to 18.7%, *P* < 0.05) was observed at 3 days after SNI (Figure 1, D–F). Notably, no elevation of mTOR activity was observed in Isolectin-B4^+^ (IB4^+^) nonpeptidergic small neurons (*P* > 0.05, Figure 1G).

By contrast, Western blot analysis of p-S6 from the SDH tissue extracts detected no difference between the contralateral and ipsilateral spinal cords following SNI (*P* > 0.05, Figure 1, H and I). Given that Western blot analysis detects the gross mTOR activity in the SDH, which may mask changes in sparsely distributed cells in the spinal cord, we carried out dual labeling of p-S6 with different cellular markers, including NeuN (neurons), GFAP (astrocytes), and Iba1 (microglia). No significant changes were observed in p-S6^+^ neurons or astrocytes between the contralateral and ipsilateral SDH within 1 week following the injury (Supplemental Figure 1, B–D). However, the number of p-S6^+^ microglia (GFP^+^) in the superficial layers of the ipsilateral SDH was robustly increased from day 3 to day 7 after SNI in *Cx3cr1^EGFP/+^* mice (*P* < 0.05, Figure 1, J and K). Together, our results demonstrate that peripheral nerve injury induces mTOR activation mainly in large DRG mechanoreceptors and SDH microglia.

### Blocking mTOR activity acutely alleviates pain.

**Figure 2:**
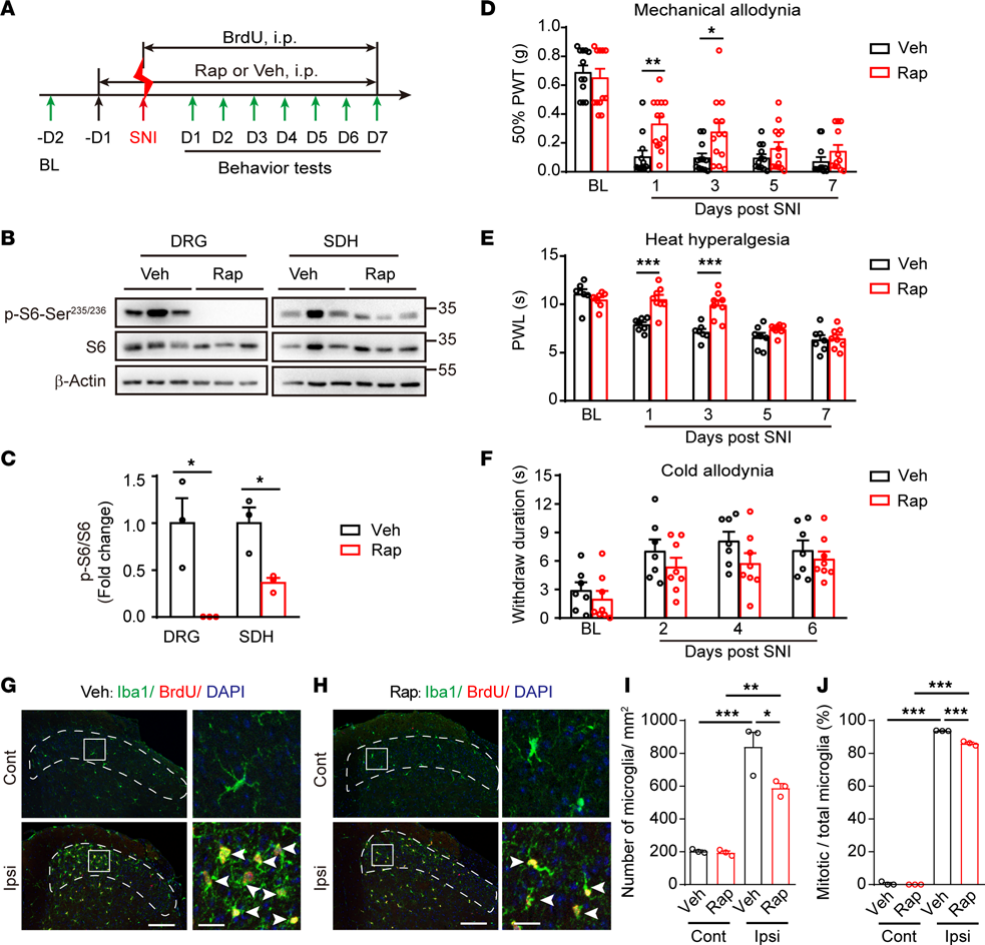
Rapamycin treatments inhibit mTOR activation and attenuate mechanical allodynia and heat hyperalgesia after SNI. (**A**) Experimental schedule for rapamycin or vehicle along with BrdU administration through intraperitoneal (i.p.) injection. (**B**) Representative blots indicating the decreased p-S6 levels in the ipsilateral DRG and SDH following 7-day continuous i.p. injection of rapamycin or vehicle in *Mtor^fl/fl^* mice after SNI. (**C**) Quantitation of p-S6/S6 in DRGs and SDH following rapamycin treatments (*n* = 3 mice per group). (**D**–**F**) Measurements of mechanical allodynia (*n* = 12–13 per group) (**D**), heat hyperalgesia (*n* =7–8 per group) (**E**), and cold allodynia (*n* = 7–8 per group) (**F**) with daily i.p. injection of rapamycin or vehicle after SNI. (**G** and **H**) Representative images of Iba1 and BrdU immunolabeling in superficial SDH (dotted regions) after treated with Veh (**G**) or rapamycin (**H**) at day 3 after SNI. Boxes show regions of higher magnification in SDH, while arrowheads indicate Iba1^+^ BrdU^+^ mitotic microglia. Scale bars: 100 and 20 μm for low- and high-magnification images, respectively. (**I** and **J**) Quantitative analysis of microglia per square millimeter (**I**) and the percentage of mitotic microglia in total microglia (**J**) in both contralateral and ipsilateral SDH at day 3 after SNI (*n* = 5–7 mice per group). Data are shown as mean ± SEM. *P < 0.05, **P < 0.01, ***P < 0.001, using 2-way ANOVA followed by Bonferroni’s post hoc tests among group (**D**–**F**), 2-tailed unpaired Student’s *t* tests (**C**), or 1-way ANOVA followed by Bonferroni’s post hoc tests (**I** and **J**). Rap, rapamycin; Veh, vehicle; BL, baseline; D, day; SDH, spinal dorsal horn; PWT, paw withdraw threshold.

To further determine the contribution of mTOR signaling in neuropathic pain, we i.p. administered rapamycin, an mTORC1 inhibitor, to systematically block the mTORC1 activity; we also administered BrdU to label proliferating cells (Figure 2A). Daily administration of rapamycin from 1 day before to 7 days after SNI significantly inhibited mTOR activity in both DRG neurons and SDH microglia (Figure 2, B and C, and Supplemental Figure 2, A–C), and it suppressed mechanical allodynia and heat hyperalgesia for the first 3 days (*P* < 0.05; Figure 2, D and E), without affecting cold allodynia (Figure 2F). Rapamycin treatments also reduced the total number of microglia (vehicle, 839.9 ± 88.3 per mm^2^; rapamycin, 588.0 ± 27.8 per mm^2^; *P* < 0.05) and the percentage of proliferative microglia (BrdU^+^ Iba1^+^) (vehicle, 93.7% ± 0.1%; rapamycin, 86.1% ± 0.7%; *P* < 0.001) in the superficial layers of ipsilateral SDH at day 3 after SNI (Figure 2, G–J). These data demonstrate that blocking mTOR signaling inhibited pain development at the acute phase and suppressed nerve damage–induced microgliosis.

### Selective ablation of mTOR in DRG neurons, but not in microglia, alleviates neuropathic pain.

**Figure 3:**
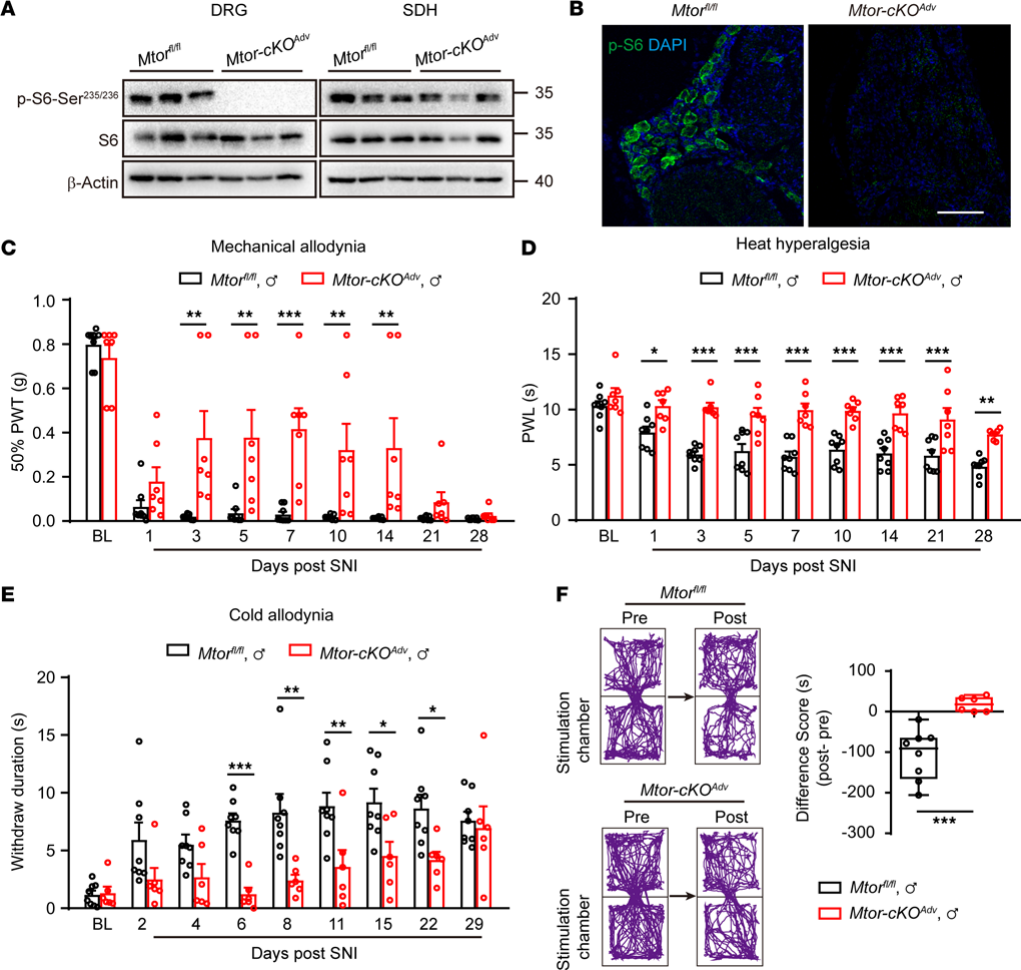
Ablation of *Mtor* in DRG neurons alleviates neuropathic pain. (**A**) Representative blots of p-S6 and S6 in the ipsilateral DRG and SDH from *Mtor^fl/fl^* and *Mtor-cKO^Adv^* mice at day 7 after SNI. (**B**) Representative images of p-S6 in the ipsilateral DRG at day 7 after SNI, indicating the ablation of mTOR in *Mtor-cKO^Adv^* mice rather than *Mtor^fl/fl^* mice after SNI. Scale bar: 100 μm. (**C**–**E**) Measurements of mechanical allodynia (**C**), heat hyperalgesia (**D**), and cold allodynia (**E**) in male *Mtor^fl/fl^* and *Mtor-cKO^Adv^* mice before and after SNI (*n* = 6–8 mice per group). (**F**) Track plots of animal movements at pre- and postconditioning phases with a 2-chamber conditioned place aversion (CPA) test (*n* = 6–8 mice per group) in male *Mtor^fl/fl^* and *Mtor-cKO^Adv^* mice at day 15 after SNI. Difference scores = postconditioning time – preconditioning time spent in the stimulation chamber. Data are shown as mean ± SEM. *P < 0.05, **P < 0.01, ***P < 0.001, by 2-way ANOVA followed by Bonferroni’s post hoc tests among groups (**C**–**E**) or 2-tailed unpaired Student’s *t* tests (**F**). BL, baseline; PWT, paw withdraw threshold; PWL, paw withdraw latency.

To further discern the contributions of neuronal or microglial mTOR in neuropathic pain, we crossed specific Cre mouse lines *Adv^cre^* or *Cx3cr1^creER^* with *Mtor^fl/fl^* mice to selectively delete the *Mtor* gene in primary sensory neurons or microglia, respectively. We observed complete elimination of p-S6 in DRG neurons and unchanged p-S6 levels in SDH in *Adv^cre^ Mtor^fl/fl^* (*Mtor-cKO^Adv^*) mice 7 days after SNI (Figure 3, A and B), demonstrating the selective ablation of mTOR in primary sensory neurons. Examination of sensory perception and motor activities found no significant differences between the control and *Mtor-cKO^Adv^* mice at basal states (Supplemental Figure 3, A–E). However, after SNI, *Mtor-cKO^Adv^* mice exhibited alleviated mechanical allodynia, heat hyperalgesia, and cold allodynia in both male and female mice for at least 14 days (Figure 3, C–E, and Supplemental Figure 3, F–H). *Mtor-cKO^Adv^* mice also had lower difference scores, representing the differences between post- and preconditioning time, in response to mechanical stimulation than the *Mtor^fl/fl^* mice in a 2-chamber conditioned place aversion (CPA) assay, which assesses the aversive responses to pain, suggesting that mTOR deletion in DRG neurons alleviated aversive responses to noxious stimuli (Figure 3F).

**Figure 4:**
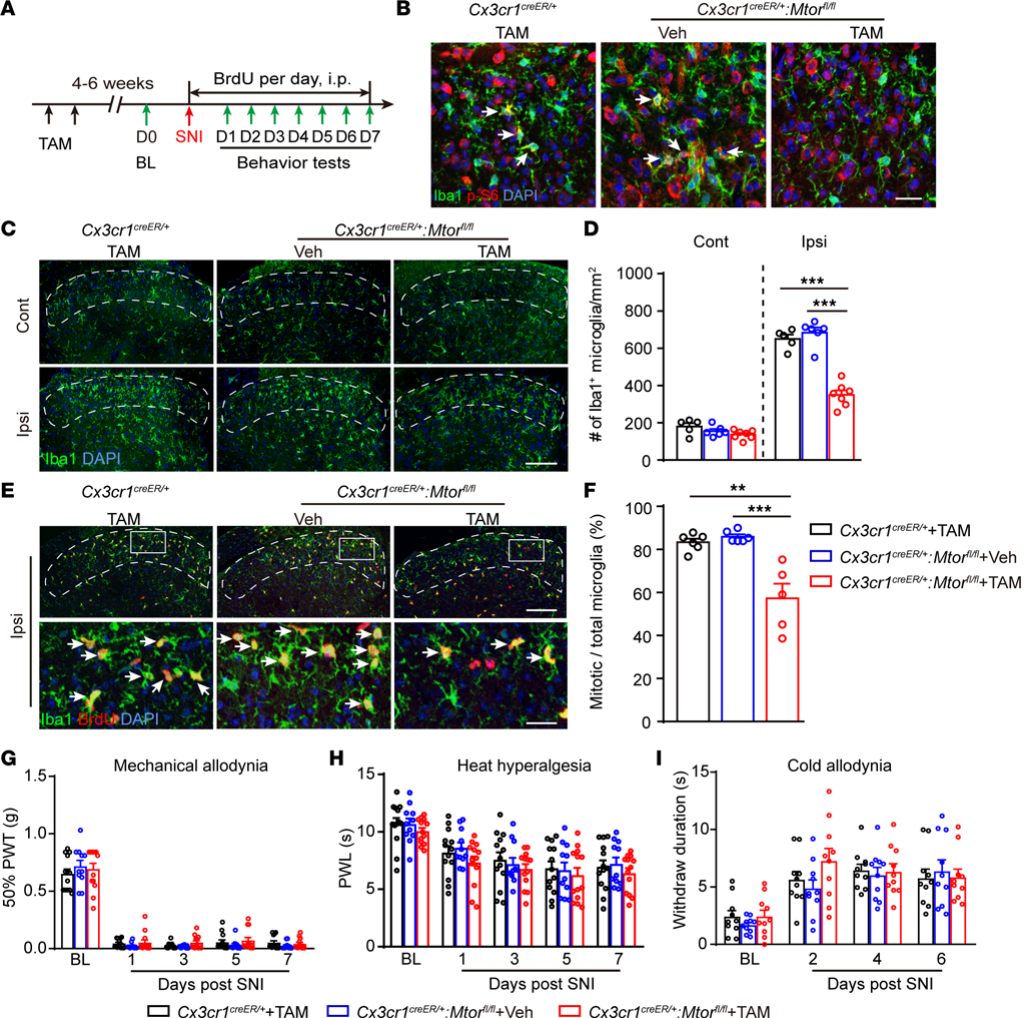
Ablation of *Mtor* in microglia reduces microgliosis but does not affect neuropathic pain in male or female mice. (**A**) Experimental schedule showing the selected *Mtor* deletion in microglia and pain tests. (**B**) Representative images showing immunofluorescence labeling of Iba1 and p-S6 in ipsilateral SDH at day 7 after SNI in *Cx3cr1^CreER/+^:Mtor^fl/fl^* or control mice (*Cx3cr1^CreER/+^* mice with TAM and *Cx3cr1^CreER/+^:Mtor^fl/fl^* mice with Veh). Arrows indicating Iba1^+^ p-S6^+^ microglia. Scale bar: 20 μm. (**C**) Representative images of bilateral SDH microglia (Iba1^+^) in *Cx3cr1^CreER/+^:Mtor^fl/fl^* mice with TAM or in control mice at day 7 after SNI. Scale bar: 100 μm. (**D**) Quantification of microglia in the ipsilateral and contralateral SDH in *Cx3cr1^CreER/+^:Mtor^fl/fl^* and control mice at day 7 after SNI (*n* = 5–7 per group). (**E**) Representative images of the ipsilateral SDH showing colocalization of Iba1 and BrdU (arrows) at day 7 after SNI. Boxes show regions of higher magnification in the SDH. Scale bars: 100 and 20 μm for low- and high-magnification images, respectively. (**F**) Quantitation of mitotic microglia (Iba1^+^BrdU^+^) in SDH in *Cx3cr1^CreER/+^:Mtor^fl/fl^* and control mice at day 7 after SNI (*n* = 5–7 mice per group). (**G**–**I**) Measurements of mechanical allodynia (**G**), heat hyperalgesia (**H**), and cold allodynia (**I**) in *Cx3cr1^CreER/+^:Mtor^fl/fl^* and control mice before and after SNI (*n* = 10–13 mice per group; male and female mice were merged). Data are shown as mean ± SEM. **P < 0.01 and ***P < 0.001, by 1-way AVOVA (**F**) or 2-way ANOVA followed by Bonferroni’s post hoc tests among groups (**D** and **G**–**I**). TAM, tamoxifen; Veh, vehicle; Cont, contralateral; Ipsi, ipsilateral; PWT, paw withdraw threshold; PWL, paw withdraw latency; D, day.

To further examine whether microglial mTOR activation also contributes to neuropathic pain, we selectively deleted *Mtor* in microglia by gavaging tamoxifen into the *Cx3cr1^creER/+^:Mtor^fl/fl^* mice (*Mtor-cKO^MG^* mice) 4–6 weeks before the SNI surgery (Figure 4A and Supplemental Figure 4A). Cre-mediated recombination of *Mtor* gene in the CNS (brain and spinal cord) was detected by PCR analysis (Supplemental Figure 4B), and reduction of p-S6 levels in the SDH microglia was verified (Figure 4B). At day 7 after SNI, the total number of microglia (Figure 4, C and D) and mitotic microglia (BrdU^+^Iba1^+^) (Figure 4, E and F) was substantially reduced in the superficial layers of ipsilateral SDH in *Mtor-cKO^MG^* mice. However, no significant differences were observed in mechanical allodynia (Figure 4G), heat hyperalgesia (Figure 4H), or cold allodynia (Figure 4I) between the *Mtor-cKO^MG^* and control mice after SNI (from day 1 to day 7), suggesting that neuropathic pain is spared in the absence of microglial mTOR signaling.

### Mtor ablation in DRG neurons suppresses elevation of subsets of nerve injury–induced genes.

**Figure 5:**
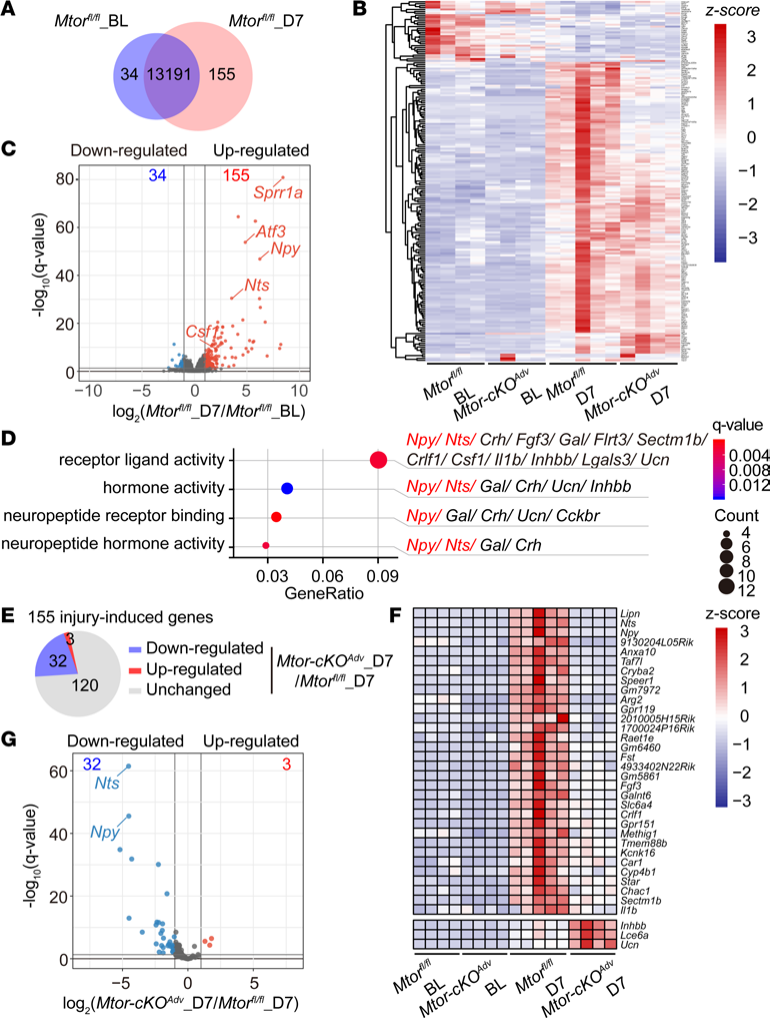
Ablation of *Mtor* in DRG neurons suppresses elevation of nerve injury–induced genes. (**A**) Venn diagram of DEGs identified in DRGs before and after SNI (day 7) in *Mtor^fl/fl^* mice (155 upregulated and 34 downregulated, *n* = 4–5 mice per group). (**B**) Heatmap of 189 DEGs by hierarchical clustering using *z* score values (*n* = 4–5 mice per group). (**C**) Volcano plots of DRG transcripts before and after SNI (day 7) in *Mtor^fl/fl^* mice. Red dots indicate 155 upregulated genes, and blue dots indicate 34 downregulated genes after SNI. (**D**) GO analysis of 155 upregulated genes after SNI and regroup into molecular function terms. All genes in each term are listed. (**E**) Pie chart of 155 injury-induced genes with 32 downregulated and 3 upregulated in *Mtor-cKO^Adv^* mice after SNI (*n* = 4–5 mice per group). (**F**) Heatmap of 35 DEGs in all samples using *z* score values. Only 3 (*Inhbb*, *Lce6a*, and *Ucn*) of the 155 injury-induced genes are upregulated upon deletion of *Mtor* in DRG neurons. (**G**) Volcano plots of 35 DEGs in control and *Mtor-cKO^Adv^* mice after SNI. Red dots indicate 3 upregulated genes, and blue dots indicate 32 downregulated genes after mTOR ablation. BL, baseline; D, day; DEGs, differentially expressed genes.

To determine the downstream molecular targets of mTOR in DRG neurons involved in neuropathic pain, we performed RNA-Seq of DRGs from *Mtor^fl/fl^* and *Mtor-cKO^Adv^* mice before and 7 days after SNI surgery. In total, the expression levels of 189 genes (155 upregulated and 34 downregulated; Supplemental Table 2) were significantly changed (by at least 2 folds, *q* < 0.05) in the injured DRGs 7 days after SNI in *Mtor^fl/fl^* mice (Figure 5, A–C). Consistent with previous studies (6, 7, 29, 30), a large number of the upregulated genes, including those associated with injury (activating transcription factor 3 [*Atf3*] and small proline-rich protein 1A [*Sprr1a*]), GPCRs (including *Gpr151* and *Gpr119*), neuropeptides (*Npy*, *Gal*, and *Nts*), cytokines (colony stimulating factor 1 [C*sf1*] and IL-1b [*Il1b*]), were identified in response to nerve injury (Figure 5B), verifying the reliability of the RNA-Seq data. Gene ontology (GO) analysis demonstrated that injury-affected genes were primarily enriched in 4 molecular functions (Figure 5D), including receptor ligand activity, hormone activity, and neuropeptide receptor binding and activity.

Importantly, approximately one-fifth (32 in 155 genes; Supplemental Table 3) of injury-induced genes were suppressed after mTOR ablation at day 7 after SNI (Figure 5E). In particular, the expression of 2 neuropeptide genes, *Npy* and *Nts*, induced by approximately 73.5 and 11.7 folds after injury, was strikingly reduced to 3.75 and 0.57 folds after ablation of *Mtor* in DRG neurons. By contrast, the expression of another 2 injury-induced neuropeptide genes, such as corticotropin releasing hormone (*Crh*) and *Gal*, remained largely unaffected, suggesting that mTOR specifically regulated the expression of subsets of injury-responsive genes (Figure 5, E–G). The reduced expression of *Npy, Nts*, and other genes (as indicated) in *Mtor-cKO^Adv^* mice was further verified by quantitative PCR (qPCR) analysis (Supplemental Figure 5). Notably, while mTOR was transiently activated during the first week after nerve injury, it may have long-term impacts through its downstream molecules. Collectively, these data demonstrate that mTOR regulates the transcription of subsets of injury-induced genes.

### Injury-activated mTOR is required for NPY induction in DRG neurons.

**Figure 6:**
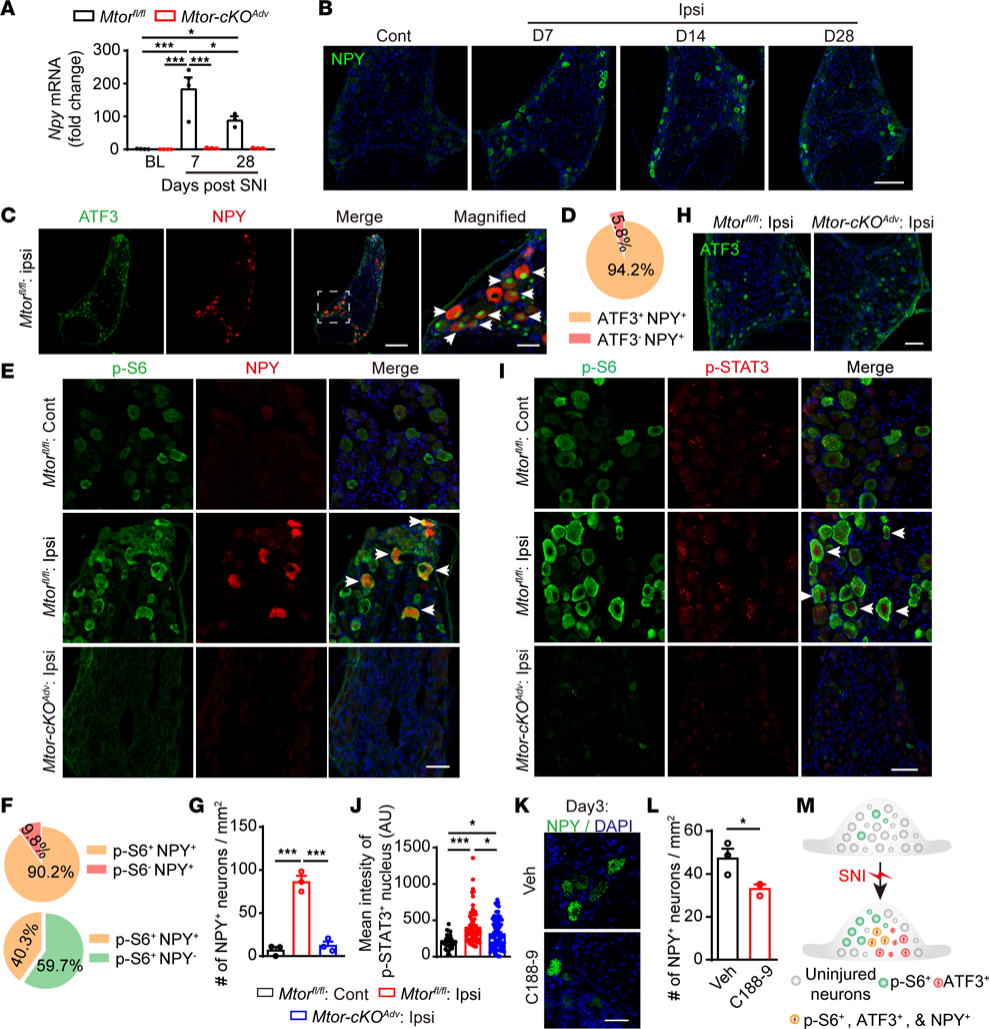
Activation of mTOR is required for NPY induction in DRG neurons after SNI. (**A**) qPCR of *Npy* in DRGs after SNI (*n* = 3–4 mice per time point). (**B**) NPY staining in DRGs from *Mtor^fl/fl^* mice. Scale bar: 100 μm. (**C**) ATF3 and NPY staining in the ipsilateral DRG from *Mtor^fl/fl^* mice at day 7 after SNI. Arrows represent colabeled neurons. Dotted box represents the region of higher magnification. Scale bars: 200 and 50 μm for low- and high-magnifications, respectively. (**D**) The ratio of NPY^+^ in ATF3^+^ neurons in the ipsilateral DRG from *Mtor^fl/fl^* mice at day 7 after SNI. (**E**) NPY and p-S6 staining in DRGs at day 7 after SNI. Arrows represent colabeled neurons. Scale bar: 50 μm. (**F**) Ratios of p-S6^+^ in NPY^+^ neurons or NPY^+^ in p-S6^+^ neurons in the ipsilateral DRG from *Mtor^fl/fl^* mice at day 7 after SNI. (**G**) Quantification of NPY^+^ neurons in *Mtor^fl/fl^* and *Mtor-cKO^Adv^* mice at day 7 after SNI (*n* = 3 mice per group). (**H**) ATF3 staining in the ipsilateral DRG at day 7 after SNI. Scale bar: 50 μm. (**I**) p-S6 and p-STAT3 staining in DRGs at day 3 after SNI. Scale bar: 50 μm. (**J**) Mean intensity of p-STAT3^+^ nucleus in DRGs at day 3 after SNI (*n* = 34, 71, and 78 cells from at least 3 mice per group). (**K** and **L**) NPY staining and quantification in the ipsilateral DRG after administration of Veh or C188-9 (*n* = 3 mice per group). Scale bar: 25 μm. (**M**) Schematic diagram showing the distribution of NPY neurons in DRGs. Data are shown as mean ± SEM. *P < 0.05 and ***P < 0.001, by 1-way ANOVA followed by Bonferroni’s post hoc tests (**A**, **G**, and **J**) or 2-tailed unpaired Student’s *t* test (**L**). Cont, contralateral; Ipsi, ipsilateral.

NPY is widely distributed in the CNS and peripheral nervous systems (31). It is absent in DRG neurons under homeostatic conditions but dramatically upregulated after peripheral nerve injury (8, 9). However, little is known about the molecular mechanisms regulating NPY induction after nerve injury. We also observed prominent induction of *Npy* in DRG neurons after nerve injury, which lasted for at least 4 weeks with gradually reduced levels after day 14 (Figure 6, A and B). Spearman correlation analysis showed that *Npy* transcripts were negatively correlated with the 50% PWT (*r* = –0.861, *P* < 0.001) and PWL (*r* = –0.865, *P* < 0.001), suggesting that the levels of *Npy* transcripts were positively correlated with pain (Supplemental Figure 6, A and B). Immunofluorescence analysis revealed that 94.2% of NPY^+^ neurons were colabeled with ATF3, a marker for injured neurons (Figure 6, C and D). Moreover, 90.2% of NPY^+^ neurons expressed p-S6, whereas only 40.3% of p-S6^+^ neurons expressed NPY (Figure 6, E and F). In addition, NPY colocalized with NF160/200 but not CGRP in the injured DRGs (Supplemental Figure 7A), suggesting that NPY was selectively induced in mTOR-activated large-sized injured neurons (Supplemental Figure 7B). Ablation of mTOR nearly eliminated NPY induction (Figure 6, E and G), indicating that mTOR was required for nerve injury–induced NPY elevation. Notably, 35.5% of p-S6^+^ neurons were ATF3^+^ and 39.8% of ATF3^+^ neurons were p-S6^+^ after peripheral nerve injury (Supplemental Figure 7, C–E), but mTOR deletion had no effects on ATF3 expression in SNI models (Figure 6H and Supplemental Figure 7C).

### mTOR promotes STAT3 phosphorylation to induce NPY production.

To further determine the potential connections between mTOR and NPY, we searched JASPAR data sets (http://jaspar.genereg.net) to identify potential binding motifs for transcriptional factors upstream of the *Npy* gene. We found several putative binding sites for Jun, cAMP-response element binding protein (CREB), and signal transducer and activator of transcription 3 (STAT3). Of note, 4 predicted binding sites of STAT3 were present in the *Npy* gene (Supplemental Table 2).

Previous studies indicate that the promoter regions of *Npy* and *Nts* genes harbor STAT3-binding site–like elements and that the dominant-negative expression of STAT3 attenuated leptin-induced *Npy* and *Nts* expression in the hypothalamus (32, 33). Moreover, nerve injury induced STAT3 phosphorylation in DRG neurons (34). Intriguingly, activated mTOR has been shown to phosphorylate STAT3 to promote its nuclear entry and downstream gene transcription (17). We, therefore, hypothesized that injury-activated mTOR may phosphorylate STAT3 to promote its nuclear entry and induce NPY transcription. To test this, we performed triple staining of NPY, p-S6, and p-STAT3, and we found that the majority of NPY^+^ neurons were also positive for both p-S6 and p-STAT3 (Figure 6M and Supplemental Figure 7F). Importantly, mTOR ablation significantly blocked the nerve injury–increased STAT3 phosphorylation and its localization in the nucleus, along with reduced NPY induction (Figure 6, I and J). Furthermore, administration of C188-9, a STAT3 inhibitor, reduced NPY induction after SNI (Figure 6, K and L), suggesting a correlation between STAT3 and NPY induction. Together, these data demonstrate that mTOR promotes NPY elevation via STAT3 phosphorylation in injured DRG neurons.

### Nerve injury–induced NPY enhances nociceptor excitability.

**Figure 7:**
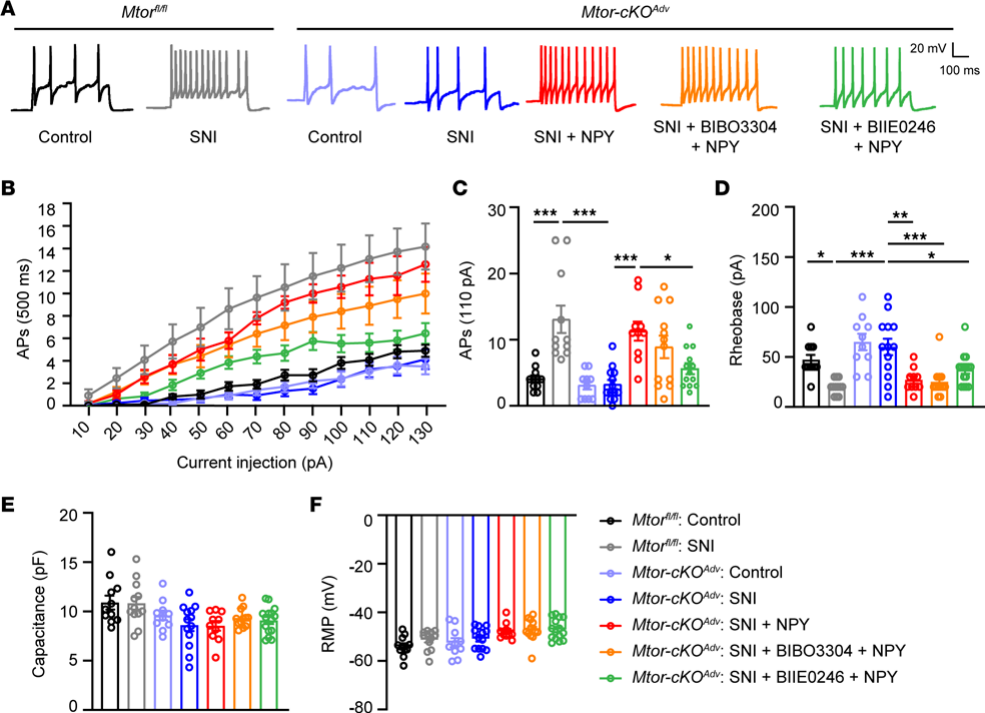
NPY enhances nociceptor excitability through Y2R. (**A**) Representative AP traces elicited by intracellular injection of 110 pA depolarizing currents on dissociated DRG neurons from resting membrane potentials (RMP) in *Mtor^fl/fl^* mice and *Mtor-cKO^Adv^* mice with or without SNI at day 7 after surgery. NPY (300 nM), BIBO3304 (1 μM), and BIIE0246 (1 μM) are replenished in medium as indicated. (**B**) The responses of *Mtor^fl/fl^* and *Mtor-cKO^Adv^* DRG neurons across a series of 500 ms depolarizing current pulses in 10 pA increment from 0 pA to 130 pA, in the presence or absence of NPY, BIBO3304, or BIIE0246 (*n* = 10–13 neurons from 3 mice per group). (**C**) Quantification of APs evoked by input current at 110 pA (*n* = 10–13 neurons from 3 mice per group). (**D**) Averaged values of rheobase currents in DRG neurons among groups measured in current-clamp (I-clamp) (*n* = 10–13 from 3 mice neurons per group). (**E** and **F**) Quantification of membrane capacitance (**E**) and RMP (**F**) among groups (*n* = 10–13 neurons from 3 mice per group). BIBO3304, Y1R antagonist; BIIE0246, Y2R antagonist. Data are shown as mean ± SEM. *P < 0.05, **P < 0.01, and ***P < 0.001, by 1-way ANOVA followed by Bonferroni’s post hoc tests among groups. AP, action potential; RMP, resting membrane potentials.

NPY has been shown to increase the excitability of DRG neurons (35). To examine whether mTOR-promoted NPY induction enhances the excitability of nociceptors, we carried out electrophysiological recording of small-sized nociceptors at 7 days after SNI. As expected, nociceptors from *Mtor^fl/fl^* mice displayed an increased number of APs and lower rheobase 7 days after SNI (Figure 7). By contrast, the number of spikes was significantly reduced in mTOR-deficient neurons. However, incubation of NPY with mTOR-deficient neurons significantly restored the number of APs and reduced the rheobase, suggesting that loss of NPY contributed to the reduced nociceptor excitability in the absence of mTOR.

Studies have demonstrated that different NPY receptor agonists elicit different responses of DRG neurons (35–37). For example, Y2R agonists increased neuronal excitability of small DRG neurons, whereas Y1R agonists barely showed any effects (35). We verified the distinct expression patterns of NPY and Y2R in DRG neurons by immunofluorescence analysis (Supplemental Figure 8A). RNA-Seq data demonstrate that *Npy2r* in DRGs was modestly increased at day 7 after SNI but decreased after *Mtor* deletion (Supplemental Figure 8B). By contrast, *Npy1r* showed no significant changes after SNI or upon mTOR ablation. To determine which receptor mediates NPY-elicited excitatory effects, we separately blocked Y1R or Y2R; we found that blocking Y2R, but not Y1R, substantially inhibited firing of DRG neurons after SNI, without affecting the rheobase, membrane capacitance, or rest potential membrane (RMP) (Supplemental Figure 8, C–H), suggesting that Y2R contributed to the increased excitability of DRG neurons after nerve injury. Moreover, blocking Y2R also prevented NPY replenishment–induced AP increase in DRG neurons, suggesting that NPY elevates neuronal excitation primarily through Y2R in DRG (Figure 7, A–C).

### Peripheral NPY replenishment reverses analgesic effects of Mtor ablation through Y2R.

**Figure 8:**
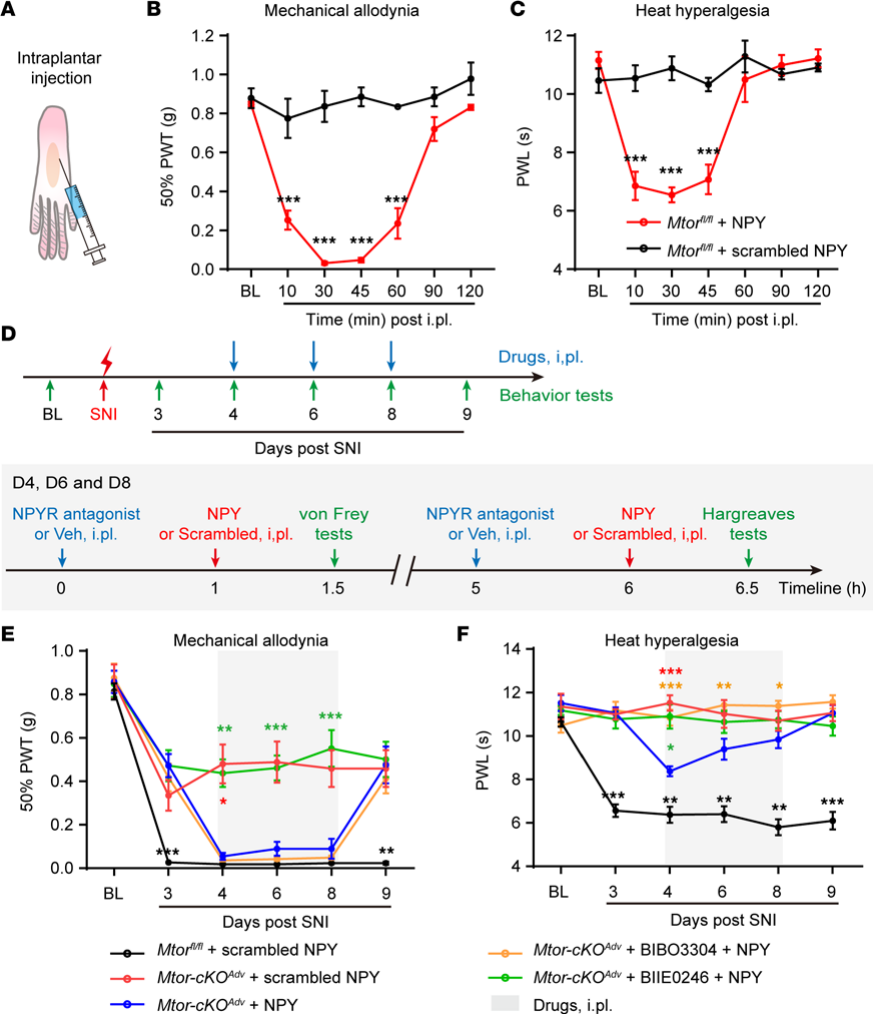
Intraplantar injection of NPY reverses analgesic effects of *Mtor* ablation through Y2R. (**A**) Schematic diagram indicating intraplantar (i.pl.) injection. (**B** and **C**) NPY (0.2 nmol) i.pl. injection into normal *Mtor^fl/fl^* mice hind paw leads to transient mechanical allodynia (**B**) and heat hyperalgesia (**C**) within an hour (*n* = 4 mice per group). (**D**) Experimental schedule showing the timeline of i.pl. injection of drugs (including NPY, NPYR antagonist, and vehicle) and behavior tests. Behavior tests were measured before and after SNI as indicated. Drugs were injected at days 4, 6, and 8. (**E** and **F**) Measurement of mechanical allodynia (**E**) and heat hyperalgesia (**F**) in *Mtor^fl/fl^* and *Mtor-cKO^Adv^* mice with i.pl. injection with NPY (0.2 nmol), scrambled NPY (0.2 nmol), BIBO3304 (5 nmol), or BIIE0246 (50 nmol) at days 4, 6, and 8 after SNI (*n* = 6–11 mice per group). BIBO3304, Y1R antagonist; BIIE0246, Y2R antagonist. Data are shown as mean ± SEM. *P < 0.05, **P < 0.01, and ***P < 0.001 versus *Mtor-cKO^Adv^* with NPY, by 2-way ANOVA followed by Bonferroni’s post hoc tests among groups. The color of asterisks indicates the statistical significance between different experimental groups (represented by different colors of labels) and *Mtor-cKO^Adv^* + NPY (shown in blue labels). BL, baseline; i.pl., intraplantar; Veh, vehicle; PWT, paw withdraw threshold; PWL, paw withdraw latency.

NPY has been shown to elicit biphasic effects in pain processing by binding to different receptors in DRG or spinal neurons (38). Given that mTOR ablation simultaneously alleviated acute pain and suppressed NPY induction, we tested whether mTOR inactivation alleviated pain via the loss of NPY. As documented before (13, 39), we first administered a small dose of NPY (0.2 nmol) into the hind paw of WT mice and observed prominent mechanical allodynia and heat hyperalgesia approximately 30 minutes after injection, supporting the pronociceptive effects of peripheral NPY administration (Figure 8, A–C). NPY injection into the *Mtor-cKO^Adv^* mice also induced robust mechanical allodynia and, to a lesser extent, heat hyperalgesia (Figure 8, D–F). Antagonizing Y2R, instead of Y1R, substantially reduced exogenous NPY-induced mechanical allodynia (Figure 8E), further supporting the role of Y2R in mediating NPY-elicited pronociceptive effects. Moreover, blocking Y2R directly inhibited pain after SNI in WT mice (Supplemental Figure 9, A and B). Collectively, our data demonstrate that mTOR-induced NPY production in DRG neurons is essential for the development of neuropathic pain via Y2R-mediated signaling.

## Discussion

Neuropathic pain is a maladaptive response of the nociceptive pathway to nerve injury. Both peripheral and central sensitization have been shown to contribute to the persistent pain (40). Peripheral nociceptor sensitization is a key trigger in neuropathic pain, since inhibiting nociceptor activity by anesthetics effectively blocks pain (40). In this study, we uncover a previously unrecognized mechanism, by which injury-induced mTOR activation drives NPY synthesis via STAT3 to enhance nociceptor excitability and promotes pain development through Y2R. Considering the distinct distribution patterns of NPY and Y2R in large mechanoreceptors and small nociceptors, mTOR-driven pain may involve intraganglionic communications between NPY-expressing mechanoreceptors and Y2R-expressing nociceptors (38).

Basal levels of mTOR activity are present in a small subset of large myelinated sensory neurons in naive mice (22, 41, 42). In the present study, we observed that increased mTOR activation predominantly occurred in large sensory neurons and spinal microglia after nerve injury. We further showed that selective ablation of mTOR in primary sensory neurons robustly prevented the early onset of nerve injury–triggered hypersensitivity for at least 2 weeks. This was in line with the temporal activation of mTOR and expression of downstream effectors in DRG neurons after nerve injury. Notably, activation of mTOR in spinal neurons remained unchanged in the current study after SNI. However, these neurons may still be able to transmit pain-related signals to the upper brain regions to mediate neuropathic pain, since blocking mTOR activities in the spinal cord, without affecting DRG, had been shown to reduce nerve–injury induced pain hypersensitivity (23). Specific ablation of mTOR in SDH neurons would be helpful to further address their function in neuropathic pain. In addition, since mTOR includes both mTOCR1 (regulatory-associated protein of mTOR, Raptor) and mTORC2 (rapamycin-insensitive companion of mTOR, Rictor), selective ablation of Raptor or Rictor in DRG neurons is needed to further distinguish their roles in neuropathic pain (43).

Nerve injury–induced de novo synthesis of a large number of molecules are implicated in the hypersensitive nociception (44–47). For example, injury-induced CSF1 in DRG neurons, a cytokine required for microglial and macrophage expansion, has recently been shown to contribute to pain hypersensitivity (29, 48). Removal of the eukaryotic initiation factor 4E-binding protein 1 (4E-BP1), a negative regulator of protein translation downstream of mTOR, induced pain hypersensitivity through enhanced translation of neuroligin 1 even in the absence of nerve injury, further stressing the importance of mTOR-mediated protein synthesis in pain hypersensitivity (49–51). Our findings that mTOR was required for nerve injury–induced NPY and NTS expression, but not ATF3, demonstrate particular links between mTOR activation and neuropeptide production. It should be noted that, although mTOR activation lasted for less than 2 weeks after nerve injury, it can trigger long-term effects by upregulating downstream molecules, such as NPY and NTS. Consistent with our data, single-cell RNA-Seq (scRNA-Seq) also detected *Npy* upregulation after peripheral nerve injury, particularly in NF1/2 subtype (A-LTMR) DRG neurons (46, 47). However, different from ours and others studies (8, 52, 53), scRNA-Seq revealed no elevation of *Npy1r* or *Npy2r* after injury (46), and this may be related to the relatively low sensitivity and unreliable detection for low-abundance transcripts by scRNA-Seq (54, 55). Moreover, scRNA-Seq shows that *Npy2r* was present in somatostatin (SST) neurons that also expressed the transient receptor potential vanilloid 1 (TRPV1), a heat sensitive channel (56, 57). It would be interesting to study whether Y2R couples with TRPV1 to promote thermal pain.

There have been efforts trying to identify molecular mechanisms underlying NPY induction. For example, nerve growth factor (NGF) appeared to increase NPY expression through an NGF-responsive element on *Npy* promoter in PC12 cells (58), whereas p-CREB–bound small heterodimer partner–interacting leucine zipper protein (SMILE) may act on *Npy* promoters to inhibit NPY expression in the hypothalamus (59). Interestingly, rapamycin treatments inhibited NPY expression in the arcuate nucleus of the hypothalamus, suggesting the involvement of mTOR in regulating NPY expression (60, 61). However, mechanisms underlying NPY induction in the injured DRG neurons remain largely unknown. Intriguingly, as a serine/threonine kinase that is primarily engaged in translational control, mTOR is unlikely to directly promote *Npy* or *Nts* transcription. In the search for potential mTOR-regulated transcriptional factors upstream of *Npy* or *Nts* genes, we observed suppressed phosphorylation of STAT3, but not c-Jun or CREB (data not shown), in DRG neurons after mTOR deletion (despite the presence of putative binding sites for all 3 transcriptional factors in the promoter region of *Npy* gene). By inhibiting STAT3 activity, we also observed reduced NPY production, suggesting that activated mTOR induced *Npy* transcription by phosphorylating STAT3, at least partially. While previous studies primarily suggest that mTOR contributes to pain sensitivity through translational control (3, 49), our study demonstrates a nontranslational mechanism of mTOR involving STAT3-NPY production in pain regulation.

NPY has been shown to elicit both antinociceptive and pronociceptive effects, depending on the subtypes of its receptors in the CNS and peripheral nervous system (38, 62). The selective induction of NPY in injured large sensory neurons suggests a peripheral effect of NPY-Y2R signaling in pain. In contrast to previous studies showing that NPY triggered analgesia by inhibiting superficial SDH interneurons through Y1R (11, 63, 64), we observed that peripheral administration of NPY promoted pain via Y2R. Previous studies show that over 40% of Y2R was expressed in CGRP neurons, and some CGRP neurons belonged to the lightly myelinated Aδ-high threshold mechanonociceptors (HTMRs) responsive to mechanical stimuli (65). Other than HTMRs, Y2R was also found in type A mechanonociceptors (AMs) responsive to nociception (14, 66). Replenishing NPY enhanced nociceptor excitability, while peripherally blocking Y2R prevented these effects, suggesting that mTOR drives NPY production to enhance nociceptor excitability through Y2R. Consistent with our observations, a previous study shows that the Y1R agonist had no effect on small DRG neurons, whereas Y2R agonist enhanced neuronal excitability (35). A possible explanation is that Y2R may attenuate calcium-sensitive potassium-conductance to induce nociceptor depolarization and excitability (35, 37). A limitation of the current study lies in the inability to directly measure NPY release from DRG, owing to the selective elevation of NPY in a particular subset of neurons. Further studies are needed to monitor NPY release and its interaction with Y2R using potentially novel fluorescent sensors (67) and to test the role of Y2R in neuropathic pain by selectively ablating Y2R in DRG neurons.

Microglia activation in SDH has been shown to contribute to neuropathic pain (68). Moreover, mTOR-mediated metabolic reprogramming was required for induction of inflammatory factors and cytokines in microglia (69), and this indicated that mTOR activation in microglia may be involved in neuropathic pain. We found that *Mtor* deletion in microglia reduced microgliosis; however, it did not have significant effects on neuropathic pain. This is likely due to the fact that mTOR was activated in less than 50% of microglia in the SDH and that mTOR ablation partially reduced microgliosis, which might not be sufficient enough to inhibit pain development. Consistent with this notion, removal of microglia only ameliorated mechanical allodynia during the first 3 days after nerve injury, whereas removal of both microglia and peripheral monocytes/macrophage prevented neuropathic pain development (30).

In summary, we demonstrate that nerve injury–induced aberrant mTOR activation in sensory neurons promotes pain development. While mTOR has been shown to affect the expression or function of hundreds of molecules, the present study links mTOR to NPY signaling in sensitizing nociceptive pathway to drive neuropathic pain. Since mTOR inhibitors are in clinical use and Y2R receptor antagonists are readily available, our findings also provide potentially new perspectives for clinically treating neuropathic pain by peripherally modulating mTOR and NPY-Y2R signaling.

## Methods

### Animals

Adult mice (8–12 weeks) of both sexes were used for biochemical and behavioral tests, and young mice (4–6 weeks) for whole-cell patch clamp recording. C57BL/6J mice were purchased from Shanghai Slac Laboratory Animal Corporation. *Cx3cr1^EGFP/+^*, *Cx3cr1^creER/+^*, *Mtor^fl/fl^*, and *Advillin^cre^* (*Adv^cre^*) mice with C57BL/6J background were purchased from The Jackson Laboratory. *Cx3cr1^EGFP/+^* mice harbor *Egfp* gene under the control of the endogenous *Cx3cr1* locus in mononuclear phagocyte system, including microglia and monocytes. *Cx3cr1^creER/+^* mice contain tamoxifen-inducible Cre recombinase elements under the direction of the *Cx3cr1* promoter. *Adv^cre^* mice displayed almost exclusive Cre-mediated recombination in all peripheral sensory neurons (70). All animals were housed under a 12-hour light/dark cycle with food and water available. To selectively KO the *Mtor* gene in microglia, mice bearing the floxed allele of the *Mtor* gene (*Mtor^fl/fl^*) were crossed with *Cx3cr1^creER/+^* mice. *Cx3cr1^creER/+^::Mtor^fl/fl^* mice received 2 doses of 10 mg tamoxifen citrate (TAM, Meilun Bio) or vehicle in 48-hour intervals. TAM induced the expression of Cre recombinase in both resident microglia and peripheral monocytes. Since monocytes have a rapid turnover rate, Cre expression is eliminated in peripheral monocytes but is maintained in resident microglia 4–6 weeks after TAM induction (71), thus allowing selective deletion of *Mtor* in microglia (*Mtor-cKO^MG^*) but not in monocytes. Control mice were *Cx3cr1^creER/+^::Mtor^fl/fl^* littermates without TAM induction and *Cx3cr1^creER/+^* mice with TAM induction. For selective ablation of *Mtor* in DRG sensory neurons, *Mtor^fl/fl^* mice were crossed with *Adv^cre^* mice to obtain the *Mtor-cKO^Adv^* mice. *Mtor-cKO^Adv^* mice enabled *Mtor* deletion in DRG neurons but leave spinal cord unaffected. Control mice were *Mtor^fl/fl^* littermates without Cre promotor.

### Antibodies and reagents

Rabbit anti–p-S6 ribosomal protein-Ser235/236 (anti–p-S6–Ser^235/236^, catalog 4858), rabbit anti–S6 ribosomal protein (catalog 2217), rabbit anti–p-STAT3–Ser^727^ (catalog 9134), and rabbit anti-NPY (catalog 11976) antibodies were all purchased from Cell Signaling Technology. Rabbit anti–NPY Y2 receptor (anti-Y2R, catalog RA14112) was purchased from Neuromics. Rat anti-BrdU (catalog ab6326), and goat anti-GFP (catalog ab5450) antibodies were obtained from Abcam. Mouse anti–β-actin (catalog A1978) and mouse anti-NF160/200 (catalog n2912) antibodies were obtained from Sigma-Aldrich. Mouse anti-GFAP (catalog 173011) antibody was purchased from Synaptic Systems (SYSY), and mouse anti-NeuN (catalog MAB377) antibody was from MilliporeSigma. Rabbit anti-Iba1 (catalog 019-19741) antibody was from Wako. Mouse anti–p-S6–Ser^235/236^ (catalog sc-293144), mouse anti-CGRP (catalog sc-57053), and mouse anti-ATF3 (catalog sc-81189) antibodies were from Santa Cruz Biotechnology Inc. The following secondary antibodies were used: Alexa Fluor 488 donkey anti-goat (Invitrogen, catalog A11055), Alexa Fluor 488 donkey anti-rabbit (Invitrogen, catalog A21206), Alexa Fluor 488 donkey anti-mouse (Invitrogen, catalog A21202), Alexa Fluor 555 donkey anti-mouse (Invitrogen, catalog A31570), Alexa Fluor 555 donkey anti-rabbit (Invitrogen, catalog A31572), and Cy3 donkey anti-rat (Jackson ImmunoResearch, catalog 712-165-153). IB4 (catalog I21412) was obtained from Invitrogen. BrdU (catalog 19-160) was obtained from Sigma-Aldrich. Rapamycin (catalog S1039) was purchased from SelleckChem. NPY (catalog 1153), scrambled NPY (catalog 3903), BIBO3304 trifluoroacetate (NPY Y1R antagonist, catalog 2412), BIIE0246 (NPY Y2R antagonist, catalog 1700), and BIIE0246 hydrochloride (catalog 7377) were purchased from Tocris.

### Cre-mediated recombination of the Mtorflox allele

Primers used for analyses of *Mtor* floxed alleles were as the following: *Mtor-P1* (5′-GCTCTTGAGGCAAATGCCACTATCACC-3′), *Mtor-P2* (5′-TCATTACCTTCTCATCAGCCAGCAGTT-3′), *Mtor-P3* (5′-TTCATTCCCTTGAAAGCCAGTCTCACC-3′). Primer pair P1/P2 was used for genotyping floxed mTOR alleles that generated a 480 bp DNA fragment in PCR (72). Upon Cre-mediated recombination, *P1/P3* pair produced a recombined *Mtor* gene fragment of 520 bp with excision of exons 1-5 (Supplemental Figure 4) (72).

### Neuropathic pain model

SNI models were used to induce neuropathic pain as previously described (73). Mice were anesthetized with sodium pentobarbital (100 mg/kg) i.p. The left hind limb was shaved, and the skin was disinfected with iodophor. After blunt separation of biceps femoris muscle, 3 distal branches of sciatic nerve were exposed, and tibia and common peroneal nerves were ligated with 5-0 silk sutures with care to avoid injury to the sural nerve. The ligated branches were then transected distal to the ligature, and a 2–3 mm distal nerve stump was removed. To minimize the number of animals used in the experiments, the right hindlimb was performed with a sham surgery after sciatic nerve exposure without nerve ligation and transection. After the surgery, the incision was closed using 5-0 silk sutures. Mice were allowed to wake up on a heating pad before being returned to their home cages. The injured side was then regarded as the ipsilateral side, and the uninjured was regarded as the contralateral one.

### Western blotting

Bilateral lumbar 4 and 5 (L4 and L5, respectively) DRGs and dorsal horns of L4 and L5 spinal cord were isolated at certain time points after SNI surgery, snap-frozen in liquid nitrogen, and stored at –80°C. Tissues were homogenized in RIPA lysis buffer (Beyotime) with protease inhibitor (catalog S8830, Sigma-Aldrich) and phosphatase inhibitor (catalog A32961, Thermo Fisher Scientific) using ultrasonic cell disruptor. The homogenates were centrifuged at 4°C for 30 minutes at 10,000*g*, and the supernatants were collected. Proteins were separated by 10% SDS-polyacrylamide gels and transferred to polyvinylidene difluoride membranes (MilliporeSigma), followed by blocking, primary antibodies, and horseradish peroxidase–conjugated (HRP-conjugated) secondary antibodies (1:10,000, Jackson ImmunoResearch) incubation. The proteins were detected using enhanced chemiluminescence (ECL) regents (Amersham Pharmacia Biotech).

### Immunofluorescence analysis

After deeply anesthetized with sodium pentobarbital, mice were perfused with saline and subsequently 4% paraformaldehyde (PFA, Sigma-Aldrich). The spinal cord and L4 and L5 DRGs were dissected, postfixed in 4% PFA, and transferred to 30% sucrose in 0.1 M phosphate buffer (pH 7.2) for 2 days. Samples were embedded in OCT (SAKURA Tissue-Tek), and transverse sections were cut using freezing microtome (Leica Biosystems) at a thickness of 15 μm. To label IB4^+^ neurons in DRGs, slices were blocked with 10% (wt/vol) normal BSA for 1 hour at room temperature, and they were incubated with 1 μg/mL IB4 diluted in PBS at room temperature for 2 hours. Sections were washed with Tris buffered saline (TBS) and then incubated with anti–p-S6 (1:1,000) antibody. For staining with other antibodies (see “Antibodies and Reagents”), sections were antigen-retrieved in citrate buffer (10 mM sodium citrate, 0.05% Tween-20, pH 6.0) or Tris-EDTA (10 mM Tris, 1 mM EDTA, 0.05% Tween-20, pH 9.0) as appropriate at 95°C for 20 minutes and permeabilized with 0.5% Triton X-100 for 10 minutes at room temperature. After blocked with 10% (wt/vol) BSA, sections were incubated overnight at 4°C with following primary antibodies: rabbit anti–p-S6 (1:1,000), mouse anti–p-S6 (1:2,000–1:4,000), mouse anti-NeuN (1:1,000), mouse anti-NF160/200 (1:2,000), mouse anti-CGRP (1:1,000), rat anti-BrdU (1:800), goat anti-GFP (1:1,000), rabbit anti-Iba1 (1:800), rabbit anti-NPY (1:1,000), mouse anti-GFAP (1:800), mouse anti-ATF3 (1:200), and rabbit anti–p-STAT3 (Ser727) (1:500). Sections were then washed in TBS with 0.5% tween (TBS-T) and incubated with appropriate secondary antibodies (1:1,000) for 1.5 hours at room temperature. For NPY and Y2R staining, since both anti-NPY and Y2R antibodies were raised in rabbits, the multiple fluorescent immunohistochemical staining kit (catalog abs50012, Absin) was used following the manufacturer instructions. The specificity of the staining using this kit was first validated by double staining of rabbit anti-NPY and Iba1 antibodies that showed no overlaps. Following the anti-NPY incubation, rabbit horseradish peroxidase–conjugated (HRP-conjugated) secondary antibody (1:1,000) was applied and incubated for 1.5 hours. Sections were than washed in TBS-T and incubated with Tyramide Signal Amplification (TSA) reagent for 10 minutes. Antibody eluent (catalog abs994, Absin) was used to wash out anti-NPY and HRP-conjugated antibodies. After washing, sections were incubated with anti-Y2R antibody (1:500) and followed by incubation with appropriate secondary antibodies (1:1,000) according to species of the first antibody. DAPI (Beyotime) was used to label cell nuclei in tissue sections. The immunofluorescence images were captured by FV-1200 confocal microscope (Olympus). The density and percentage of positive cells in SDH and DRGs were counted and calculated using 3 sections from each animal. Mean intensity of interested regions were evaluated using ImageJ software (NIH). Relative mean intensity of p-STAT3 staining in the cell nuclei was calculated as mean intensity in the nucleus subtracted that in the cytoplasm.

### Drug administration

BrdU was used to label proliferating cells in the spinal cord after the SNI surgery. The BrdU labeling procedure was carried out as described before (74), with 2 i.p. injections (100 mg/kg) daily 1 day before the surgery until 7 days after surgery. For i.p. treatment of rapamycin, mice were administrated with rapamycin (5 mg/kg) or vehicle daily 1 day before SNI until 7 days after surgery. For local intraplantar (i.pl.) injection, drugs (0.2 nmol NPY, 0.2 nmol scrambled NPY, 5 nmol BIBO3304 trifluoroacetate, or 50 nmol BIIE0246) in 20 μL saline were injected using a syringe with a 30 gauge needle. Dosages of NPY and its antagonists were referred to the previous studies (12, 13). NPY receptor antagonists were injected 1 hour before NPY injection. To assess the effects after i.pl. injection, behavioral tests were finished in 30–40 minutes after NPY or scrambled peptide injection. The von Frey and Hargreaves tests were used for an interval of at least 4 hours. For i.p. treatment of STAT3 inhibitor, mice were administrated with 5 mg/kg C188-9 (S8605, SelleckChem) or vehicle daily from 1 day before SNI to 3 days after SNI.

### RNA sequencing

Bilateral SNI were performed in *Mtor^fl/fl^* and *Mtor-cKO^Adv^* mice to minimize the animals used in the experiment. In total, 4 lumber DRGs (bilateral L4 and L5 DRGs) were collected from each mouse before or 7 days after SNI. RNAs were isolated using RNeasy micro kit (catalog 74004, QIAGEN) according to the manufacturer’s instructions. RNA-Seq libraries were constructed and sequenced by BGISEQ-500 (BGI). After quality control, the raw RNA-Seq data were filtered to obtain the clean data used for alignment to the mouse genome (Mus musculus GRCm38.p5, NCBI). Based on these read counts, normalization and assessment of differential gene expression were performed using DESeq2 on *R* (version 3.5.3). Genes with fragments per kilobase million lower than 1 (FPKM < 1) in all groups were excluded from the subsequent analyses. Statistical significance of differentially expressed genes (DEGs) was calculated based on the raw counts of individual genes, with an absolute fold change greater than 2 and adjusted *P* value (equivalent to *q* value) less than 0.05.

Volcano plots and heatmaps were visualized by R (the ggplot2 and gplots packages, respectively). GO enrichment in the molecular function category was visualized by R (bioconductor package “org.Hs.eg.db” and “cluster profiler” package).

### qPCR

Total RNA from DRGs was extracted using RNeasy micro kit and reverse transcribed using PrimeScript RT Reagent Kit (catalog RR037A, TaKaRa). Real-time PCR was performed using the SYBR Premix Ex Taq (catalog DRR041A, Takara) on a LightCycler 480 Instrument II Real-Time PCR Detection System (Roche). Primer sequences are provided in Supplemental Table 1. The relative expression was measured using the 2^−ΔΔCt^ method. Briefly, the Ct values of target genes were determined automatically by LightCycler 480 II software. ΔCt = Ct_(Target_ _genes)_ – Ct_β-actin_. ΔΔCt = ΔCt_(Target_ _genes)_ − ΔCt_(average_ _ΔCt_ _of control)_. Relative fold changes were determined by 2^−ΔΔCt^ and normalized to the expression levels of *Actin* (75).

### Whole-cell patch clamp recording

Mice were anesthetized with sodium pentobarbital before being sterilized with 75% alcohol. L4 and L5 DRGs were carefully collected on ice and digested with collagenase IV (0.2 mg/mL, catalog LS004188, Worthington Biochemical) and dispase-II (3 mg/mL, catalog D4693, Sigma-Aldrich) for 60 minutes at 37°C. The cell suspension was centrifuged at 500*g* for 10 minutes through a cushion of 15% BSA (catalog A9205, Sigma-Aldrich) in order to eliminate most of the cellular debris. The cell pellet was resuspended in Neurobasal medium (catalog 21103049, Thermo Fisher Scientific) with B27 (catalog 17504-044, Invitrogen, Thermo Fisher Scientific) and NGF supplement (50 ng/mL, catalog 13257-019, Gibco, Thermo Fisher Scientific), seeded onto glass coverslips coated with poly-D-lysine (catalog P7280, Sigma-Aldrich), and cultured in 5% CO_2_ incubator at 37°C for at least 2 hours before recording. For drug treatment, cultured DRG neurons were incubated with 300 nM NPY for 30 minutes before recording. To antagonize Y1R or Y2R, BIBO3304 trifluoroacetate (BIBO3304, 1 μM) or BIIE0246 hydrochloride (1 μM) was replenished, respectively, into medium 30 minutes before NPY addition.

Whole-cell patch clamp recordings were carried out at room temperature using a Multiclamp 700B amplifier (Molecular Devices). Small DRG neurons (< 20 μm and membrane capacitance < 70 pF) with healthy appearance (rounded smooth edges and no apparent nucleus) were selected for further recording. After gigaohm (GΩ) seal and membrane ruptured, the RMP was monitored until it is stabilized. The neurons with resting potential more positive than –40 mV were discarded. APs were evoked by current injection steps. The resistances of borosilicate glass electrodes were measured ranging from 3 to 5 MΩ. The intracellular pipette solution contained (in mM) 135 K-gluconate, 6 NaCl, 10 HEPES, 0.5 EGTA, 10 Na_2_-phosphocreatine, 4 Mg-ATP, and 0.3 Na_2_-GTP and was adjusted to pH 7.2 using KOH. The extracellular solution was composed of (in mM) 150 NaCl, 5 KCl, 2.5 CaCl_2_, 1 MgCl_2_, 10 HEPES, and 10 glucose and was adjusted to pH 7.4 by NaOH. AP firing and RMP were recorded from small-diameter neurons. Data were collected from neurons with stable RMP more negative than –40 mV. APs were evoked by current injection steps. Data were digitized with Digidata 1440A (Molecular Devices) and analyzed by *pClamp* software (Version 10.6, Molecular Devices).

### Behavioral tests

The following behavioral tests were conducted in a blinded manner and during daytime (light cycle). For all experiments, experimenters were blinded to genotypes or experimental manipulation. All the apparatuses and cages were sequentially wiped with 70% ethanol and ddH_2_O; they were then air-dried between stages.

#### von Frey tests.

von Frey tests were used to evaluate 50% paw withdrawal threshold (50% PWT) during the light cycle. In brief, individual mice were habituated in an opaque plexiglas chamber on a wire mesh platform for 30 minutes prior to testing. Testing was performed using a set of von Frey filaments (0.008–2 g, North Coast Medical). Each filament was applied to the lateral part of plantar surface of the mouse hind paw vertically for up to 3 seconds from the bottom. Positive response was determined as a sharp withdrawal, shaking, or licking of the limb. The 50% PWT was determined by the up-down method (76).

#### Hargreaves tests.

Thermal sensitivity was examined using Hargreaves radiant heat apparatus (IITC Life Science). The basal paw withdrawal latency was adjusted to 9–12 seconds, with a cutoff of 20 seconds to avoid tissue damage.

#### Hot plate tests.

Mice were placed on the hot plate (IITC Life Science) at 50°C, 52°C, or 56°C, and the reaction time was scored when the animal began to exhibit signs of pain avoidance such as jumping or paw licking. Animals that did not respond to the noxious heat stimulus after 40 seconds were removed from the plate.

#### Acetone tests.

For cold allodynia assay, a 20 μL drop of acetone was gently applied to the hind paw surface using a syringe fitted with a blunted needle. Researches avoided mechanical stimulation of the paw with the syringe during application. The total duration of paw withdrawal, defined as the total time of flinching, licking, or biting of the limb, was recorded with a maximum cutoff time of 60 seconds.

#### Rotarod tests.

A rotarod system (Panlab, Spain) was used to assess motor function. Mice were tested in 3 separated trials with a 10-minute interval. During the tests, the speed of rotation was accelerated from 4 to 40 rpm over a 5-minute period. The falling latency was recorded.

#### Open field tests.

Mice were placed in the middle of a novel open field arena (45 cm length × 45 cm width × 30 cm height) under normal light conditions. Using ANY-maze software (Stoelting), the distance the animal walked in 10 minutes was recorded.

#### CPA tests.

CPA experiments were conducted in a 2-chamber device (50 × 25 cm) at day 15 after SNI. The CPA protocol included preconditioning (baseline), conditioning, and postconditioning phases (10 min during each phase). Animals spending > 500 seconds or < 100 seconds of the total time in either chamber in the preconditioning phase were eliminated from further analysis. Immediately following the preconditioning phase, the mice underwent conditioning for 10 minutes. During conditioning, 1 of the 2 chambers was paired with the mechanical stimuli. The mechanical stimulus was repeated every 10 seconds with a 0.16 g von Frey filament on the left hind paw when the mouse entered into the condition chamber. During the postconditioning phase, the animals did not receive any stimuli and had free access to both compartments for a total of 10 minutes. Animal movements in each of the chambers were recorded, and the time spent in both chambers was analyzed using ANY-maze software. Difference scores were defined as postconditioning time subtracted from preconditioning time spent in the stimuli-paired chamber.

### Prediction of transcriptional factors of *Npy*

The JASPAR database was employed to predict the potential binding targets of *Npy* gene. First, the sequence extending from 2,000 bp upstream to 100 bp downstream of the transcription start site of the *Npy* gene was downloaded from the NCBI database (https://www.ncbi.nlm.nih.gov/). Thereafter, this sequence was compared with the transcriptional factors in the JASPAR database with a relative profile score threshold of 80%.

### Data availability

Sequencing data have been deposited in GEO under accession code GSE184014, which includes all data generated or analyzed during this study.

### Statistics

Statistical analyses were performed using GraphPad Prism (Version 8.0.1). Quantitative measurements are presented as mean ± SEM. Measurements that lie outside 2 SD were excluded. Statistical differences in comparison with the control group were analyzed using 2-tailed paired or unpaired *t* tests as appropriate. One-way (for multiple comparisons) or 2-way ANOVA (for multiple time points) with Bonferroni’s post hoc tests were used for experiments with more than 2 groups. Spearman correlation analysis was performed to show correlations between *Npy* transcripts and behavior measurements. Significance was considered with *P* < 0.05. Regarding replication, every mouse represents a replicate, and the number of replicates and additional information on statistics (sample sizes, tests, and *P* values) are mentioned for each experiment in the figure legend.

### Study approval

All experiments were conducted in the Zhejiang University School of Medicine. The study protocol and the use and care of animals were reviewed and approved by The Tab of Animal Experimental Ethical Inspection of the First Affiliated Hospital, College of Medicine, Zhejiang University (no. 2017054).

## Author contributions

ZG, LC, YH, YW, and SW designed experiments and wrote the manuscript. LC, YH, SW, KC, WM, and WS performed experiments and analyzed the data. ZG, LC, YH, YW, SD, YJG, LHZ, ZZX, and HM modified the paper. ZG and YW supervised the project.

## Supplementary Material

Supplemental data

Supplemental table 3

## Version 1 10/04/2022

In-Press Preview

## Version 2 11/22/2022

Electronic publication

## References

[1] Van Hecke O (2014). Neuropathic pain in the general population: a systematic review of epidemiological studies. Pain.

[2] Costigan M (2009). Neuropathic pain: a maladaptive response of the nervous system to damage. Annu Rev Neurosci.

[3] Melemedjian OK, Khoutorsky A (2015). Translational control of chronic pain. Prog Mol Biol Transl Sci.

[4] Geppetti P (2015). G protein-coupled receptors: dynamic machines for signaling pain and itch. Neuron.

[5] Xia L-P (2021). GPR151 in nociceptors modulates neuropathic pain via regulating P2X3 function and microglial activation. Brain.

[6] Reinhold AK (2015). Differential transcriptional profiling of damaged and intact adjacent dorsal root ganglia neurons in neuropathic pain. PLoS One.

[7] Wu S (2016). Dorsal root ganglion transcriptome analysis following peripheral nerve injury in mice. Mol Pain.

[8] Xiao HS (2002). Identification of gene expression profile of dorsal root ganglion in the rat peripheral axotomy model of neuropathic pain. Proc Natl Acad Sci U S A.

[9] Wakisaka S (1991). Increased neuropeptide Y (NPY)-like immunoreactivity in rat sensory neurons following peripheral axotomy. Neurosci Lett.

[10] Solway B (2011). Tonic inhibition of chronic pain by neuropeptide Y. Proc Natl Acad Sci U S A.

[11] Nelson TS, Taylor BK (2021). Targeting spinal neuropeptide Y1 receptor-expressing interneurons to alleviate chronic pain and itch. Prog Neurobiol.

[12] Sapunar D (2011). Attenuation of pain-related behavior evoked by injury through blockade of neuropeptide Y Y2 receptor. Pain.

[13] Tracey DJ (1995). Peripheral hyperalgesia in experimental neuropathy: exacerbation by neuropeptide Y. Brain Res.

[14] Arcourt A (2017). Touch receptor-derived sensory information alleviates acute pain signaling and fine-tunes nociceptive reflex coordination. Neuron.

[15] Saxton RA, Sabatini DM (2017). mTOR signaling in growth, metabolism, and disease. Cell.

[16] Carlin D (2018). Deletion of Tsc2 in nociceptors reduces target innervation, ion channel expression, and sensitivity to heat. eNeuro.

[17] Laplante M, Sabatini DM (2013). Regulation of mTORC1 and its impact on gene expression at a glance. J Cell Sci.

[18] Abe N (2010). Mammalian target of rapamycin (mTOR) activation increases axonal growth capacity of injured peripheral nerves. J Biol Chem.

[19] Zhang W (2013). Activation of mTOR in the spinal cord is required for pain hypersensitivity induced by chronic constriction injury in mice. Pharmacol Biochem Behav.

[20] Xu JT (2014). Opioid receptor-triggered spinal mTORC1 activation contributes to morphine tolerance and hyperalgesia. J Clin Invest.

[21] Melemedjian OK (2011). Targeting adenosine monophosphate-activated protein kinase (AMPK) in preclinical models reveals a potential mechanism for the treatment of neuropathic pain. Mol Pain.

[22] Geranton SM (2009). A rapamycin-sensitive signaling pathway is essential for the full expression of persistent pain states. J Neurosci.

[23] Asante CO (2010). Mammalian target of rapamycin signaling in the spinal cord is required for neuronal plasticity and behavioral hypersensitivity associated with neuropathy in the rat. J Pain.

[24] Obara I (2011). Systemic inhibition of the mammalian target of rapamycin (mTOR) pathway reduces neuropathic pain in mice. Pain.

[25] Tateda S (2017). Rapamycin suppresses microglial activation and reduces the development of neuropathic pain after spinal cord injury. J Orthop Res.

[26] Norsted Gregory E (2010). Mammalian target of rapamycin in spinal cord neurons mediates hypersensitivity induced by peripheral inflammation. Neuroscience.

[27] Xu Q (2011). Spinal phosphinositide 3-kinase-Akt-mammalian target of rapamycin signaling cascades in inflammation-induced hyperalgesia. J Neurosci.

[28] Melemedjian OK (2013). mTORC1 inhibition induces pain via IRS-1-dependent feedback activation of ERK. Pain.

[29] Guan Z (2016). Injured sensory neuron-derived CSF1 induces microglial proliferation and DAP12-dependent pain. Nat Neurosci.

[30] Peng J (2016). Microglia and monocytes synergistically promote the transition from acute to chronic pain after nerve injury. Nat Commun.

[31] Allen Y (1983). Neuropeptide Y distribution in the rat brain. Science.

[32] Muraoka O (2003). Leptin-induced transactivation of NPY gene promoter mediated by JAK1, JAK2 and STAT3 in the neural cell lines. Neurochem Int.

[33] Cui H (2005). Anorexigenic hormones leptin, insulin, and alpha-melanocyte-stimulating hormone directly induce neurotensin (NT) gene expression in novel NT-expressing cell models. J Neurosci.

[34] Chen WT (2016). Rapamycin-resistant mTOR activity is required for sensory axon regeneration induced by a conditioning lesion. eNeuro.

[35] Abdulla FA, Smith PA (1999). Nerve injury increases an excitatory action of neuropeptide Y and Y2-agonists on dorsal root ganglion neurons. Neuroscience.

[36] Wiley JW (1993). Agonists for neuropeptide Y receptor subtypes NPY-1 and NPY-2 have opposite actions on rat nodose neuron calcium currents. J Neurophysiol.

[37] Abdulla FA, Smith PA (1999). Neuropeptide Y actions and the distribution of Ca2+-dependent Cl- conductance in rat dorsal root ganglion neurons. J Auton Nerv Syst.

[38] Brumovsky P (2007). Neuropeptide tyrosine and pain. Trends Pharmacol Sci.

[39] Brumovsky P (2005). Neuropeptide Y2 receptor protein is present in peptidergic and nonpeptidergic primary sensory neurons of the mouse. J Comp Neurol.

[40] Colloca L (2017). Neuropathic pain. Nat Rev Dis Primers.

[41] Lisi L (2015). mTOR kinase: a possible pharmacological target in the management of chronic pain. Biomed Res Int.

[42] Harris J (2008). Local translation in primary afferent fibers regulates nociception. PLoS One.

[43] Laplante M, Sabatini DM (2012). mTOR signaling in growth control and disease. Cell.

[44] Zhao JY (2017). DNA methyltransferase DNMT3a contributes to neuropathic pain by repressing Kcna2 in primary afferent neurons. Nat Commun.

[45] Wang K (2021). Single-cell transcriptomic analysis of somatosensory neurons uncovers temporal development of neuropathic pain. Cell Res.

[46] Renthal W (2020). Transcriptional reprogramming of distinct peripheral sensory neuron subtypes after axonal injury. Neuron.

[47] Kupari J (2021). Single cell transcriptomics of primate sensory neurons identifies cell types associated with chronic pain. Nat Commun.

[48] Lu X Microglia and macrophages contribute to the development and maintenance of sciatica in lumbar disc herniation. Pain.

[49] Khoutorsky A, Price TJ (2018). Translational control mechanisms in persistent pain. Trends Neurosci.

[50] Khoutorsky A (2015). Translational control of nociception via 4E-binding protein 1. Elife.

[51] Yousuf MS (2020). Pharmacological manipulation of translation as a therapeutic target for chronic pain. Pharmacol Rev.

[52] Zhang X (1997). Expression and regulation of the neuropeptide Y Y2 receptor in sensory and autonomic ganglia. Proc Natl Acad Sci U S A.

[53] Landry M (2000). Effect of axotomy on expression of NPY, galanin, and NPY Y1 and Y2 receptors in dorsal root ganglia and the superior cervical ganglion studied with double-labeling in situ hybridization and immunohistochemistry. Exp Neurol.

[54] Sarkar A, Stephens M (2021). Separating measurement and expression models clarifies confusion in single-cell RNA sequencing analysis. Nat Genet.

[55] Potter SS (2018). Single-cell RNA sequencing for the study of development, physiology and disease. Nat Rev Nephrol.

[56] Usoskin D (2015). Unbiased classification of sensory neuron types by large-scale single-cell RNA sequencing. Nat Neurosci.

[57] Caterina MJ (1999). A capsaicin-receptor homologue with a high threshold for noxious heat. Nature.

[58] Minthworby CA (1994). Transcriptional regulation of the human neuropeptide-Y gene by nerve growth-factor. J Biol Chem.

[59] Lv X (2021). Skeleton interoception regulates bone and fat metabolism through hypothalamic neuroendocrine NPY. Elife.

[60] Oh TS (2016). Hypothalamic AMPK-induced autophagy increases food intake by regulating NPY and POMC expression. Autophagy.

[61] Varela L (2012). Hypothalamic mTOR pathway mediates thyroid hormone-induced hyperphagia in hyperthyroidism. J Pathol.

[62] Diaz-delCastillo M (2018). Neuropeptide Y and its involvement in chronic pain. Neuroscience.

[63] Taiwo OB, Taylor BK (2002). Antihyperalgesic effects of intrathecal neuropeptide Y during inflammation are mediated by Y1 receptors. Pain.

[64] Miyakawa A (2005). Action of neuropeptide Y on nociceptive transmission in substantia gelatinosa of the adult rat spinal dorsal horn. Neuroscience.

[65] Ghitani N (2017). Specialized mechanosensory nociceptors mediating rapid responses to hair pull. Neuron.

[66] Hill RZ, Bautista DM (2020). Getting in touch with mechanical pain mechanisms. Trends Neurosci.

[67] Wu Z (2022). Pushing the frontiers: tools for monitoring neurotransmitters and neuromodulators. Nat Rev Neurosci.

[68] Inoue K, Tsuda M (2018). Microglia in neuropathic pain: cellular and molecular mechanisms and therapeutic potential. Nat Rev Neurosci.

[69] Hu Y (2019). mTOR-mediated metabolic reprogramming shapes distinct microglia functions in response to lipopolysaccharide and ATP. Glia.

[70] Zurborg S (2011). Generation and characterization of an Advillin-Cre driver mouse line. Mol Pain.

[71] Parkhurst CN (2013). Microglia promote learning-dependent synapse formation through brain-derived neurotrophic factor. Cell.

[72] Risson V (2009). Muscle inactivation of mTOR causes metabolic and dystrophin defects leading to severe myopathy. J Cell Biol.

[73] Decosterd I, Woolf CJ (2000). Spared nerve injury: an animal model of persistent peripheral neuropathic pain. Pain.

[74] Gu N (2016). Spinal microgliosis due to resident microglial proliferation is required for pain hypersensitivity after peripheral nerve injury. Cell Rep.

[75] Livak KJ, Schmittgen TD (2001). Analysis of relative gene expression data using real-time quantitative PCR and the 2(−Delta Delta C(T))method. Methods.

[76] Dixon WJ (1965). The up-and-down method for small samples. J Am Stat Assoc.

