# Supplementary material for: mTOR–neuropeptide Y signaling sensitizes nociceptors to drive neuropathic pain
Source: JCI Insight. 2022 Nov 22;7(22):e159247. doi: 10.1172/jci.insight.159247 (PMC9746821; doi:10.1172/jci.insight.159247)
Supplement: Supplemental data [file jciinsight-7-159247-s032.pdf]

Supplemental material

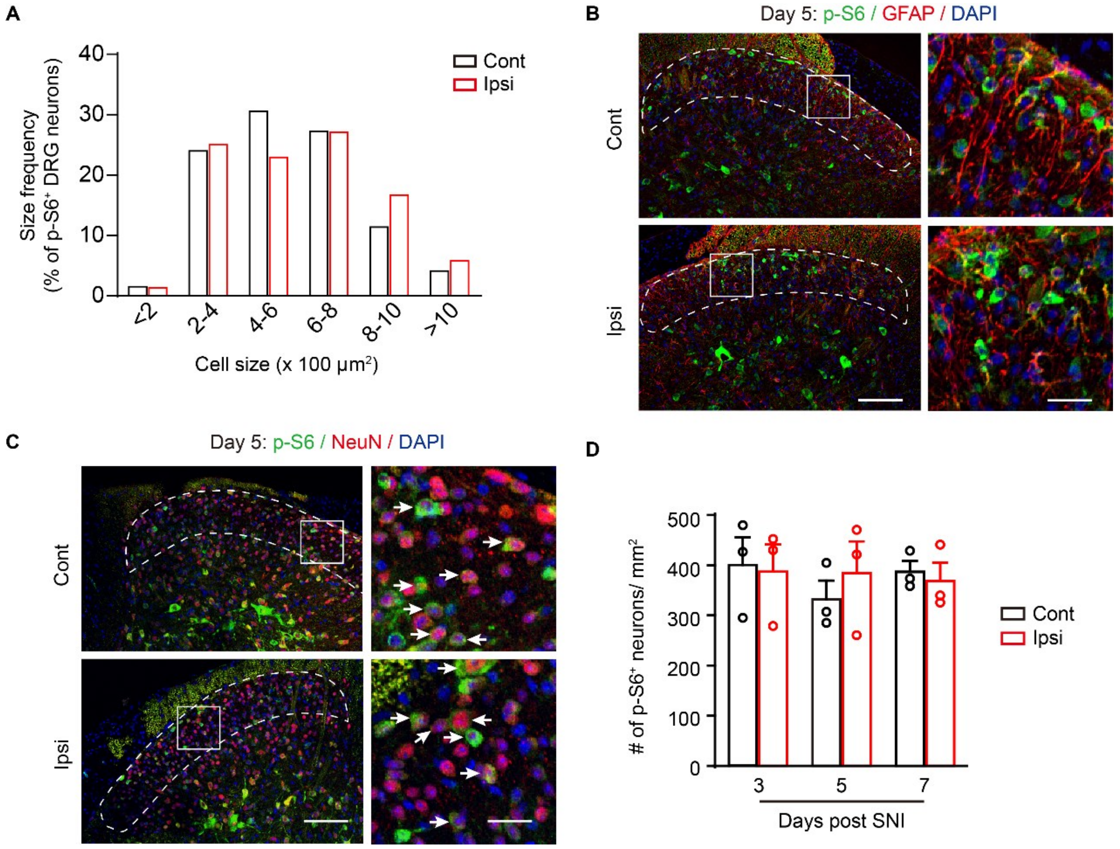

Supplementary Figure 1. Characterizing of p-S6 staining with different markers in DRGs or SDH after SNI. (**A**) Size frequency of p-$S6^+$ neurons from contralateral and ipsilateral DRGs at day 3 post SNI (n=579 and 718 neurons from 3-4 mice respectively). (**B**) Representative images of p-S6 and NeuN immunolabeling (arrows) in superficial SDH (dotted regions) at day 5 after SNI. Boxes show the region with magnification. Scale bars, 100 μm and 20 μm for lower- and higher-magnification images, respectively. (**C**) Quantitation of p-$S6^+$ neurons in superficial SDH at day 3 to 7 after SNI (n=3 mice per time point). Values are means ± SEM. Two-way ANOVA followed by Bonferroni's *post hoc* tests among groups. Scale bars, 100 μm and 20 μm for lower- and higher-magnification images, respectively. Ipsi, ipsilateral; Cont, contralateral. (**D**) Representative images of p-S6 and GFAP in superficial SDH (dotted regions) at day 5 after SNI. Boxes show the region with magnification.

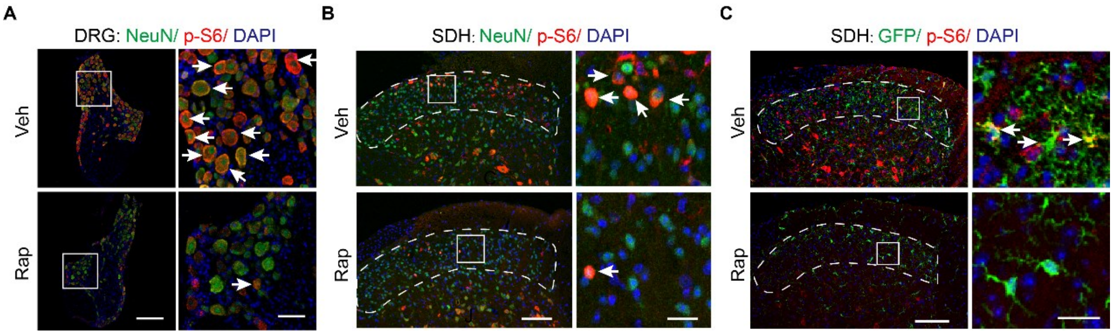

Supplementary Figure 2. Administration of rapamycin suppresses mTOR activation in DRGs and spinal cord. (**A**-**C**) Co-immunostaining of p-S6 with NeuN or GFP in the ipsilateral DRG (**A**) and SDH (**B**, **C**) after i.p. administration of vehicle or rapamycin (arrows indicating p-S6$^+$ cells) at day 7 after SNI. Boxes show regions with magnification. Scale bars, 100 μm and 20 μm in (**A**), and 200 μm and 50 μm in (**B**, **C**) for lower- and higher-magnification images, respectively. Rap, rapamycin; Veh, vehicle; BL, baseline; SDH, spinal dorsal horn.

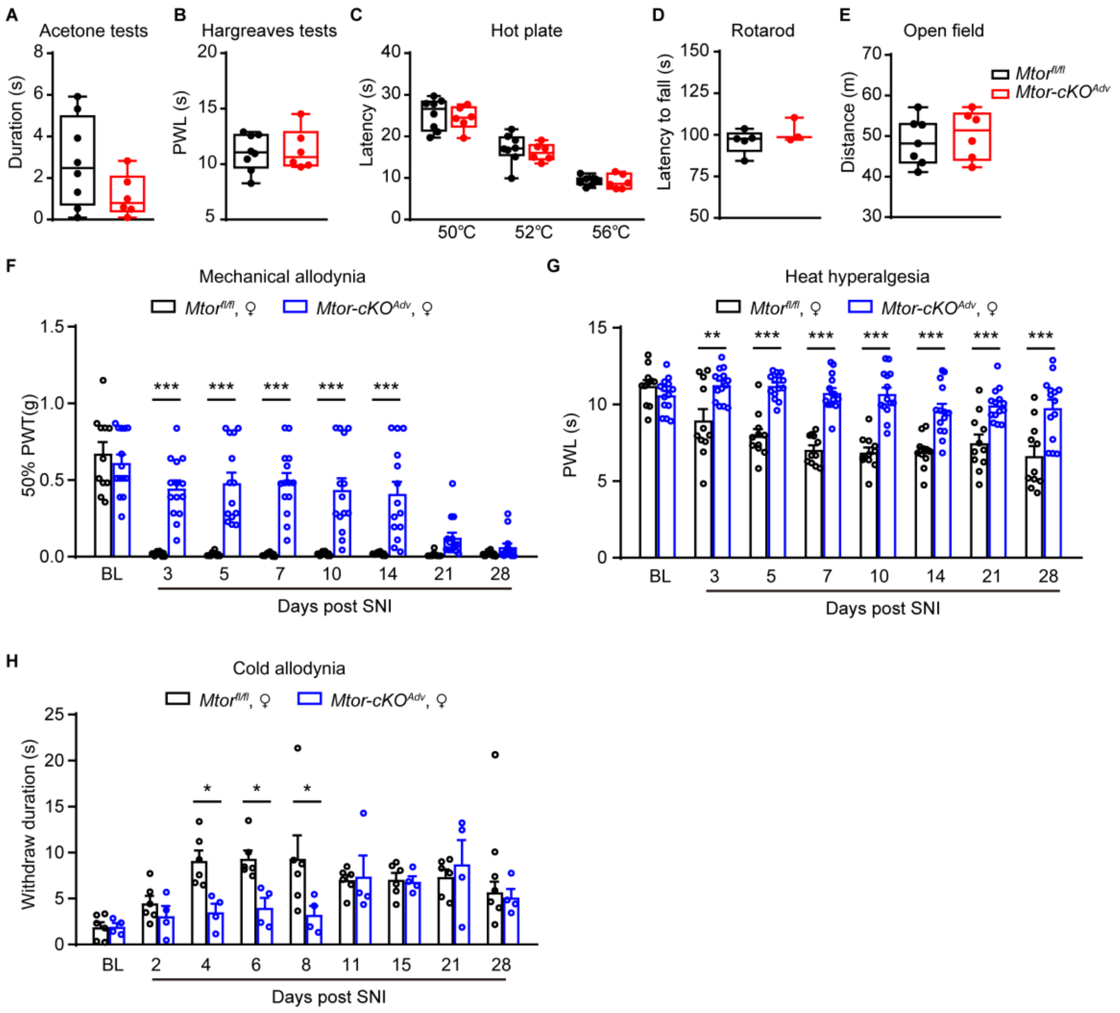

Supplementary Figure 3. Measurements of basal sensory functions, motor activities, and pain-like behaviors after SNI in female *Mtor$^{fl/fl}$* and *Mtor-cKO^{Adv}* mice. (**A**) Acetone tests (n=6-8 mice per group). (**B**) Hargreaves tests (n=6-8 mice per group). (**C**) Hot plate tests (n=6-8 mice per group). (**D**) Rotarod tests (n=3-5 mice per group). (**E**) Open field tests in *Mtor$^{fl/fl}$* and *Mtor-cKO^{Adv}* mice (n=6-7 mice per group). (**F-H**) Measurements of mechanical allodynia (**F**) and heat hyperalgesia (**G**) in female *Mtor$^{fl/fl}$* (n=11) and *Mtor-cKO^{Adv}* mice (n=14) before and after SNI. (**H**) Measurements of cold allodynia in female *Mtor$^{fl/fl}$* (n=6) and *Mtor-cKO^{Adv}* mice (n=4). Values are means ± SEM. * p<0.05, ** p<0.01, *** p<0.001, 2-tailed unpaired student t-tests (**A, B, D, E**) and two-way ANOVA followed by Bonferroni's *post hoc* tests among groups (**C, F, G, H**).

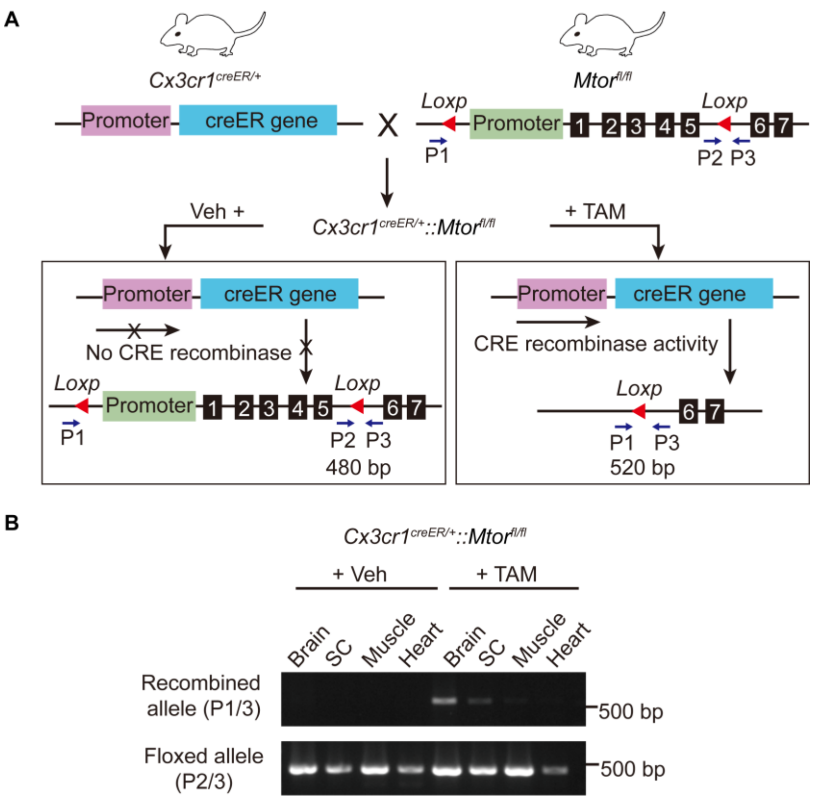

Supplementary Figure 4. Stratagem for generating *Mtor-cKO*^{MG} mice. (A) Schematic showing the generation of *Mtor-cKO*^{MG} mice. Exons 1-5 of the *Mtor* gene is flanked by *loxP* sites and excised in microglia expressing Cx3cr1-Cre recombinase after TAM administration. The position of P1, P2 and P3 primers and the size of the DNA segments amplified by primer pairs are illustrated. (**B**) Agarose gel electrophoresis of P1, P2 and P3 PCR products showing that Cre-mediated recombination is specifically occurred in the central nervous system (brain and spinal cord), but not in other peripheral tissues (muscle or heart). TAM, tamoxifen; Veh, vehicle.

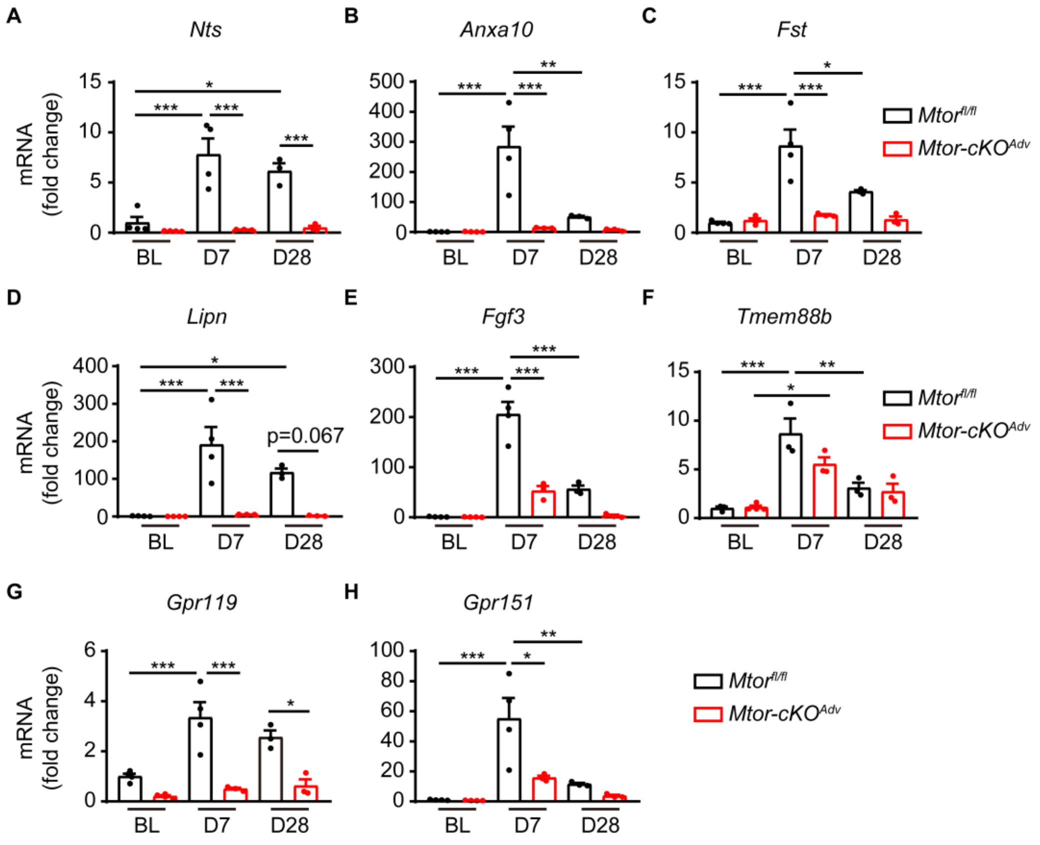

Supplementary Figure 5. Quantitative RT-PCR of downregulated DEGs identified in RNA sequencing. (**A**) *Nts*, neurotensin; (**B**) *Anxa10*, annexin A10; (**C**) *Fst*, follistatin; (**D**) *Lipn*, lipase family member N; (**E**) *Fgf3*, fibroblast growth factor 3; (**F**) *Tmem88b*, transmembrane protein 88b; (**G**) *Gpr119*, G protein-coupled receptor 119; (**H**) *Gpr151*. n=3-4 mice per time point per group. * p<0.05, ** p<0.01, *** p<0.001, one-way ANOVA followed by Bonferroni's *post hoc* tests among groups. BL, baseline; D, day; DEGs, differentially expressed genes.

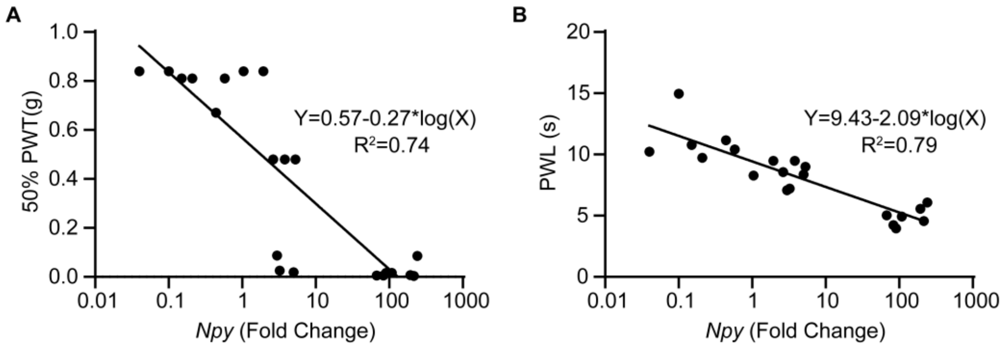

Supplementary Figure 6. Spearman correlation analysis between *Npy* transcripts and pain behavioral tests. (**A**) von Frey tests. (**B**) Hargreaves tests. Semilog lines with equation are illustrated.

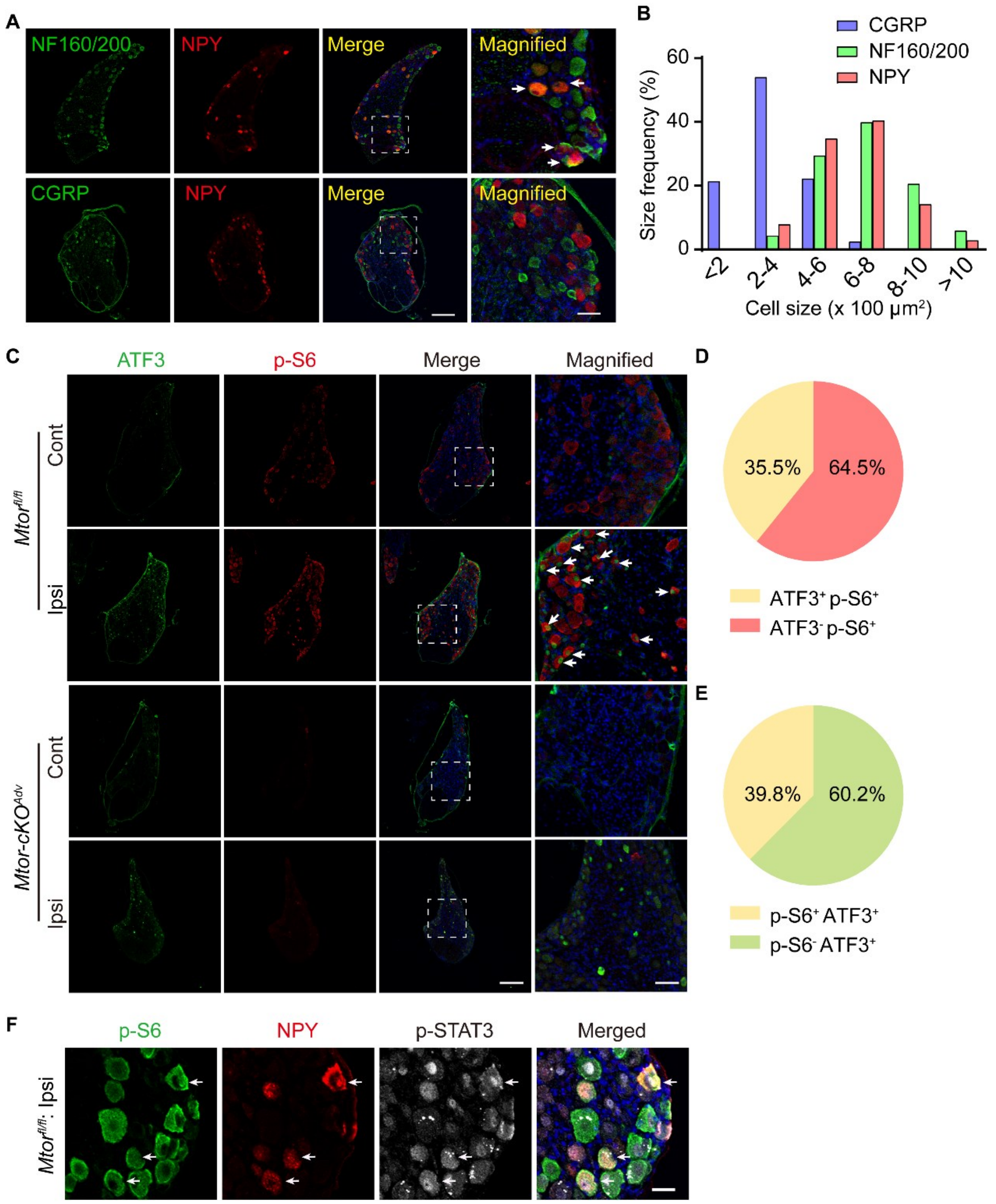

Supplementary Figure 7. Ablation of mTOR does not reduce neuronal injury after SNI. (**A**) Representative images of NPY and NF160/200 or CGRP staining in the injured DRGs. Arrows indicating both NPY^+ and $NF160/200^+$ neurons. Boxes show regions with magnification. Scale bars, 200 μm and 50 μm for lower- and higher-magnification images, respectively. (**B**) Size frequency of $CGRP^+$, $NF160/200^+$, and NPY^+ neurons in DRGs post SNI. (**C**) Representative images showing immunofluorescence labeling of ATF3 and p-S6 in contralateral and ipsilateral DRGs

from *Mtor*$^{fl/fl}$ and *Mtor-cKO*^{Adv} mice. Arrows indicating ATF3^{+} p-S6^{+} neurons. Boxes show regions with magnification. Scale bars, 200 μm and 50 μm for lower- and higher-magnification images, respectively. (**D-E**) Pie charts indicating the ratio of ATF3^{+} neurons in total p-S6^{+} neurons (**D**) or p-S6^{+} neurons in total ATF3^{+} neurons (**E**) in the ipsilateral DRG at day 7 after SNI. Cont, contralateral; Ipsi, Ipsilateral. (**F**) Representative images showing immunofluorescence labeling of p-S6, NPY and p-STAT3 in the ipsilateral DRG.

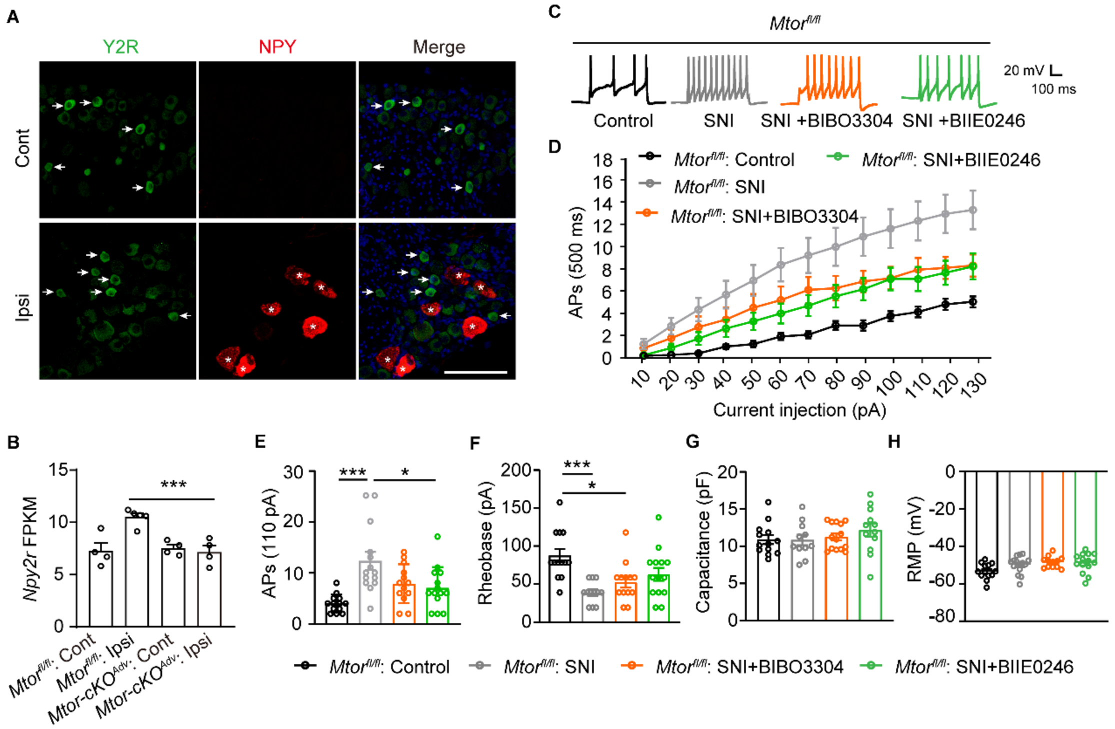

Supplementary Figure 8. NPY receptor antagonists modestly inhibit the DRG neuronal excitability after SNI. (**A**) Distinct expression pattern of NPY (*) and Y2R (arrows) by immunofluorescence analysis at day 7 after SNI. Scale bar, 100 μm. (**B**) Fragments per kilobase million (FPKM) of *Npy2r* in contralateral and ipsilateral DRGs from $Mtor^{fl/fl}$ and $Mtor\text{-}cKO^{Adv}$ mice at day 7 after SNI. *** *q-value*<0.001, statistical significance of differentially expressed genes (DEGs) was calculated based on the raw counts of individual genes using DESeq2 on *R*. (**C**) Representative AP traces were elicited by intracellular injection of 110 pA depolarizing currents on dissociated DRG neurons from resting membrane potentials (RMP) in $Mtor^{fl/fl}$ mice with or without SNI. BIBO3304 and BIIE0246 are replenished in the medium as indicated. (**D**) The response of $Mtor^{fl/fl}$ DRG neurons across a series of 500 ms depolarizing current pulses in 10 pA increment from 0 pA to 130 pA, in the presence or absence of BIBO3304 or BIIE0246 (n=10-13 neurons from 4 mice). (**E**) Quantification of APs evoked by input current at 110 pA (n=10-13 neurons from 4 mice). (**F**) Averaged values of rheobase currents in DRG neurons among groups measured in I-clamp (n=10-13 neurons from 4 mice). (**G-H**) Quantification of membrane capacitance (**G**) and RMP (**H**) among groups (n=10-13 neurons from 4 mice). BIBO3304, Y1R antagonist; BIIE0246, Y2R antagonist.

Values are means ± SEM. * $p<0.05$, ** $p<0.01$, and *** $p<0.001$, one-way ANOVA followed by Bonferroni's *post hoc* tests among groups. AP, action potential; RMP, resting membrane potentials.

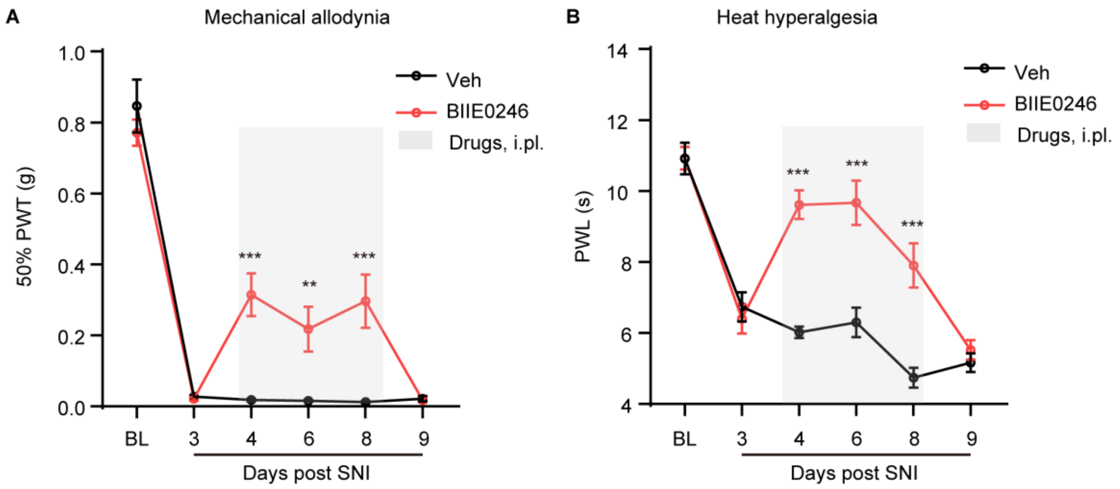

Supplementary Figure 9. Intraplantar injection of Y2R antagonists alleviated neuropathic pain after SNI. (**A-B**) Measurement of mechanical allodynia (**A**) and heat hyperalgesia (**B**) in C57BL/6J mice with i.pl. injection with Veh or BIIE0246 (50 nmol) at day 4, 6, and 8 after SNI (n = 8 mice per group). Values are means ± SEM. ** $p<0.01$, and *** $p<0.001$ *vs.* Veh injection, two-way ANOVA followed by Bonferroni's *post hoc* tests among groups. BL, baseline; i.pl., intraplantar; Veh, vehicle; BIIE0246, Y2R antagonist; PWT, paw withdraw threshold; PWL, paw withdraw latency.

Supplementary Table 1. Primer sequences in RT-PCR.

Gene name	Forward	Reverse
Nts	CCTGACTCTCCTGGCTTTCA	CCGGGCTGTTCACGTTATTT
Npy	GGACTGACCCTCGCTCTATC	CTTCAAGCCTTGTTCTGGGG
Annexin a10	CATCCTAACACAACGCAGCA	AGTTCCTGGTCCCTTCATGG
Lipn	AAGTTCGGAAGTCCTCTGGG	GAATCCACCAGCGCTTAAGG
Gpr119	GTCACTATCAGCCATCCGGA	GCTGGCCGACTTCTAGAGAT
Fst	CGAATGTGCACTCCTCAAGG	ACTGTTCAGAAGAGGAGGGC
Gpr151	GTATGGCATGTGAAGGCTGG	GCCTCCTGAACCTCTGAAGT
Fgf3	ACCTGGCCATGAACAAGAGA	ACACGTACCAAGGTCTCTGG
Csf1	TGCTAAGTGCTCTAGCCGAG	CCCCCAACAGTCAGCAAGAC
Actin	GTGACGTTGACATCCGTAAAGA	GCCGGACTCATCGTACTCC

Supplementary Table 2. Prediction of STAT3 binding sites at -2000 bp – 100 bp of the *Npy* gene

Matrix ID	Name	Score	Relative score	Start	End	Strand	Predicted sequence
MA0144.1	MA0144.1.Stat3	12.19	0.91	379	388	+	TTTCAGGAAG
MA0144.1	MA0144.1.Stat3	7.05	0.82	118	127	+	GTCCAGGAGG
MA0144.1	MA0144.1.Stat3	6.48	0.81	527	536	+	GTCCAGGAGG
MA0144.1	MA0144.1.Stat3	6.32	0.80	1161	1170	-	CCCCTGGAAG
